# Electrostatic Potential Complementarity for Thickness‐Tolerant Cathode Interlayers in High‐Efficiency Organic and Tandem Solar Cells

**DOI:** 10.1002/advs.75134

**Published:** 2026-04-02

**Authors:** Xiaoman Ding, Dana Mukasheva, Jiaxu Che, Guangye Zhang, Xiuze Hei, Haoran Lin, Jie Lv, Patrick Fong, Zhiwei Ren, Annie Ng, Mingjian Yuan, Hongyu Zhang, Gang Li, Hanlin Hu

**Affiliations:** ^1^ Hoffmann Institute of Advanced Materials Shenzhen Polytechnic University Shenzhen China; ^2^ State Key Laboratory of Supramolecular Structure and Materials College of Chemistry Jilin University Changchun China; ^3^ Department of Electrical and Computer Engineering School of Engineering and Digital Sciences Nazarbayev University Research Administration Nazarbayev University Astana 010000 Kazakhstan; ^4^ College of New Materials and New Energies Shenzhen Technology University Shenzhen China; ^5^ Department of Electrical and Electronic Engineering Research Institute for Smart Energy (RISE) Photonic Research Institute (PRI) The Hong Kong Polytechnic University Kowloon Hong Kong SAR China; ^6^ College of Chemistry Nankai University Tianjin China

**Keywords:** cathode interlayer, electrostatic potential, intermolecular interactions, organic solar cells, thickness tolerance

## Abstract

Designing cathode interlayers (CILs) that simultaneously deliver high efficiency, operational stability, and broad thickness tolerance remains a critical challenge for organic solar cells (OSCs). Herein, trimesic acid (TMA) and phloroglucinol (PG), two small molecules featuring opposite central electrostatic potential (ESP) distributions, are employed as model systems to systematically elucidate their intermolecular interactions with the benchmark CIL material PDINN and to reveal the molecular origin of thickness sensitivity. Benefiting from complementary ESP matching, PG incorporation markedly enhances PDINN self‐doping, electrical conductivity, energy‐level alignment, and *π*–*π* stacking, while suppressing PDINN self‐agglomeration and reducing Ag electrode work function. These synergistic effects promote efficient charge extraction and transport and suppress non‐radiative recombination losses. As a result, OSCs with the PDINN:PG CIL achieve a power conversion efficiencie (PCE) of 20.0% (certified 19.5%), together with outstanding thickness tolerance, maintaining 87.0% of champion efficiency at 50 nm, and improved operational stability (80% after 600 h). Furthermore, perovskite‐organic tandem solar cells (TSCs) deliver a decent PCE of 26.4%. This work establishes an ESP‐guided molecular interfacial engineering strategy for thickness‐tolerant and durable CILs for next‐generation OSCs.

## Graphical abstract

Two model molecules — trimesic acid (TMA) and phloroglucinol (PG) — with opposite central electrostatic potential (ESP) distributions are employed to elucidate how molecular electrostatic interactions regulate the cathode interlayer (CIL) PDINN. Electrostatic potential complementarity is revealed as the intrinsic origin of thickness sensitivity in CILs, enabling simultaneous enhancement of thickness tolerance and photovoltaic performance in organic solar cells (OSCs).

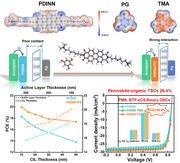

## 1 Introduction

Organic solar cells (OSCs), recognized as one of the most promising photovoltaic (PV) technologies with power conversion efficiencies (PCEs) surpassing 21%, exhibit compelling commercial application prospects through inherent merits including mechanical flexibility, lightweight nature, solution processability, and chemically versatile structures enabling tailored material design [1, 2, 3, 4, 5, 6, 7, 8, 9, 10, 11, 12, 13]. Although studies in recent years have reported progress in the large‐area fabrication of OSCs, their device efficiencies still lag behind laboratory‐scale counterparts. Beyond the challenges in morphology control arising from the enlarged active layer area, ultrathin interfacial layers (5–10 nm), particularly cathode interfacial layers (CILs), often prove incompatible with large‐scale processing requirements [14, 15, 16]. Therefore, there is an urgent need to develop CILs with low thickness sensitivity to meet the demands of future industrial‐scale production.

Effective CILs typically require high electron affinity and high electron mobility to enhance device photovoltaic performance and operational stability. In recent developments of OSCs, n‐type self‐doped organic semiconductors have emerged as the dominant CIL materials [17, 18]. These include conjugated polymer electrolytes such as amine‐functionalized polyfluorene derivatives (Poly [(9,9‐bis (3'‐ (N,N‐dimethylamino)propyl)‐2,7‐fluorene)‐alt‐2,7‐ (9,9–dioctylfluorene) (PFN) [19], Poly[(9,9‐bis(3'‐(N,N‐dimethyl)‐N‐ethylammoniumpropyl‐2,7‐fluorene)‐alt‐2,7‐(9,9‐dioctylfluorene)] bromide (PFN‐Br) [20]), naphthalene diimide (NDI) derivatives (poly[(9,9‐bis(3′‐(N,N‐dimethylamino)propyl)‐2,7‐fluorene)‐alt‐5,5′‐bis(2,2′‐thiophene)‐2,6‐naphthalene‐1,4,5,8‐tetracaboxylic‐N,N′‐di(2‐ethylhexyl)imide] (PNDIT‐F3N) [21], Humidity‐resistance cathode interlayer material (NDIP3F‐M) [22]), and perylene diimide (PDI) derivatives (N,N’‐bis[3‐(dimethylamino)propyl]perylene‐3,4,9,10‐tetracarboxylic diimide (PDIN) [23], N,N′‐Bisperylene‐3,4,9,10‐tetracarboxylic diimide (PDINN) [24]). These materials incorporate polar/ionic functional groups into their molecular structures, strengthening interactions with the active layer interface through non‐covalent forces (e.g., hydrogen bonding and *π*–*π* stacking). This improves thin‐film morphology, lowers the electrode work function, and facilitates ohmic contact [14, 24, 25, 26]. However, the presence of polar/ionic side chains can detrimentally affect the ordered molecular packing of these CILs [27, 28]. Moreover, their optimal charge extraction and interfacial dipole effects are constrained to a narrow processing window of just a few nanometers. This limitation compromises electron transport and conductivity, leading to pronounced thickness sensitivity and poor device stability. To address these challenges, Bo et al. [29] developed an amide‐functionalized PDI‐Leu‐am molecule as a CIL. This material simultaneously enables electron extraction and work function reduction while inhibiting acceptor degradation. Although the resulting device achieved a PCE of 20% with significantly enhanced stability under light and thermal stress, the thickness sensitivity issue remained unresolved. Consequently, the design strategy for CIL materials faces a critical need to reconcile high conductivity with low thickness sensitivity, potentially requiring significant trade‐offs. For instance, Li and colleagues [30]. designed a composite interfacial layer by blending PDINN with multi‐fluorinated copper phthalocyanine (CuPc). The synergistic effects arising from hydrogen bonding and *π*–*π* interactions enhanced conductivity and improved interfacial properties, yielding an OSC based on PM6: D18: L8‐BO with a PCE of 20.17%. This system also demonstrated excellent thickness tolerance and versatility. Similarly, Huang et al. [4] constructed an organic/inorganic composite interface (A‐ZnO‐F3N), effectively leveraging the synergy between 2D A‐ZnO and PNDIT‐F3N. This approach significantly reduced interfacial defect density while boosting interfacial conductivity and film uniformity, achieving a PCE of 20.6% for a D18: L8‐BO‐based OSC alongside remarkably enhanced thickness tolerance and device stability. Despite these advances, achieving high‐performance composite CILs often relies on serendipitous blending through extensive trial and error. Fundamental gaps persist: the systematic realization of CILs combining high conductivity with low thickness sensitivity remains elusive, and a foundational understanding of molecular‐level property control is lacking. This knowledge gap impedes the further optimization of device efficiency and stability, as well as the establishment of reliable design principles for high‐performance CIL materials. While our previous studies demonstrated that hydroxyl groups regulate film morphology through hydrogen bonding and carboxyl groups improve the thick‐film tolerance of CILs via acid‐base interactions that facilitate self‐doping, the individual contributions of these functional groups remain unclear [17, 18].

Building on this premise, we deliberately selected two well‐defined model molecules—trimesic acid (TMA) and phloroglucinol (PG)—to selectively probe hydrogen bonding and acid–base interactions, respectively. These two molecules exhibit opposing molecular electrostatic potential (ESP) distributions at their core, providing an ideal platform for mechanistic comparison. The design enables a systematic investigation into how these interactions govern the intermolecular forces with the CIL material PDINN, thereby elucidating their distinct contributions to the performance regulation of thick‐film CILs. Combined theoretical calculations and experimental analyses reveal that the complementary ESP matching and strengthened intermolecular interactions between PG and PDINN effectively modulate the physicochemical properties of the interlayer. Specifically, PG incorporation significantly enhances PDINN self‐doping and electrical conductivity, optimizes energy level alignment and molecular *π*–*π* stacking order, suppresses PDINN self‐agglomeration, and reduces the Ag electrode work function. These synergistic effects facilitate efficient charge extraction and transport while simultaneously suppressing bimolecular and trap‐assisted recombination, thereby minimizing non‐radiative energy loss. Consequently, OSCs employing the PDINN:PG CIL achieve a decent PCE of 20.0%, markedly outperforming devices based on pristine PDINN (18.3%) and PDINN:TMA (19.1%). Moreover, PDINN:PG also integrates excellent thickness tolerance (87.0% of optimal PCE at 50 nm) and enhanced operational stability, with an unencapsulated device retaining 80% of its initial PCE after 600 h of continuous light illumination using an LED solar simulator (SLS‐LED‐80) in a nitrogen environment at ambient temperature. Notably, this strategy demonstrates broad generality, being compatible with diverse mainstream active layer systems (e.g., PM6: N3, PM6: Y6, PM6: BO‐4Cl, PM6: L8‐BO) and compatible with conventional CIL materials (e.g., PFN‐Br, NDI‐ph, and PNDIT‐F3N). Furthermore, in perovskite‐organic tandem solar cells (TSCs), these interfacial advantages are further amplified, delivering a high PCE of 26.4%. This work establishes a robust molecular interfacial engineering strategy and theoretical framework for designing high‐performance, stable CILs, providing critical insights toward performance optimization and practical deployment of OSC technologies.

## 2 Results and Discussion

**FIGURE 1:**
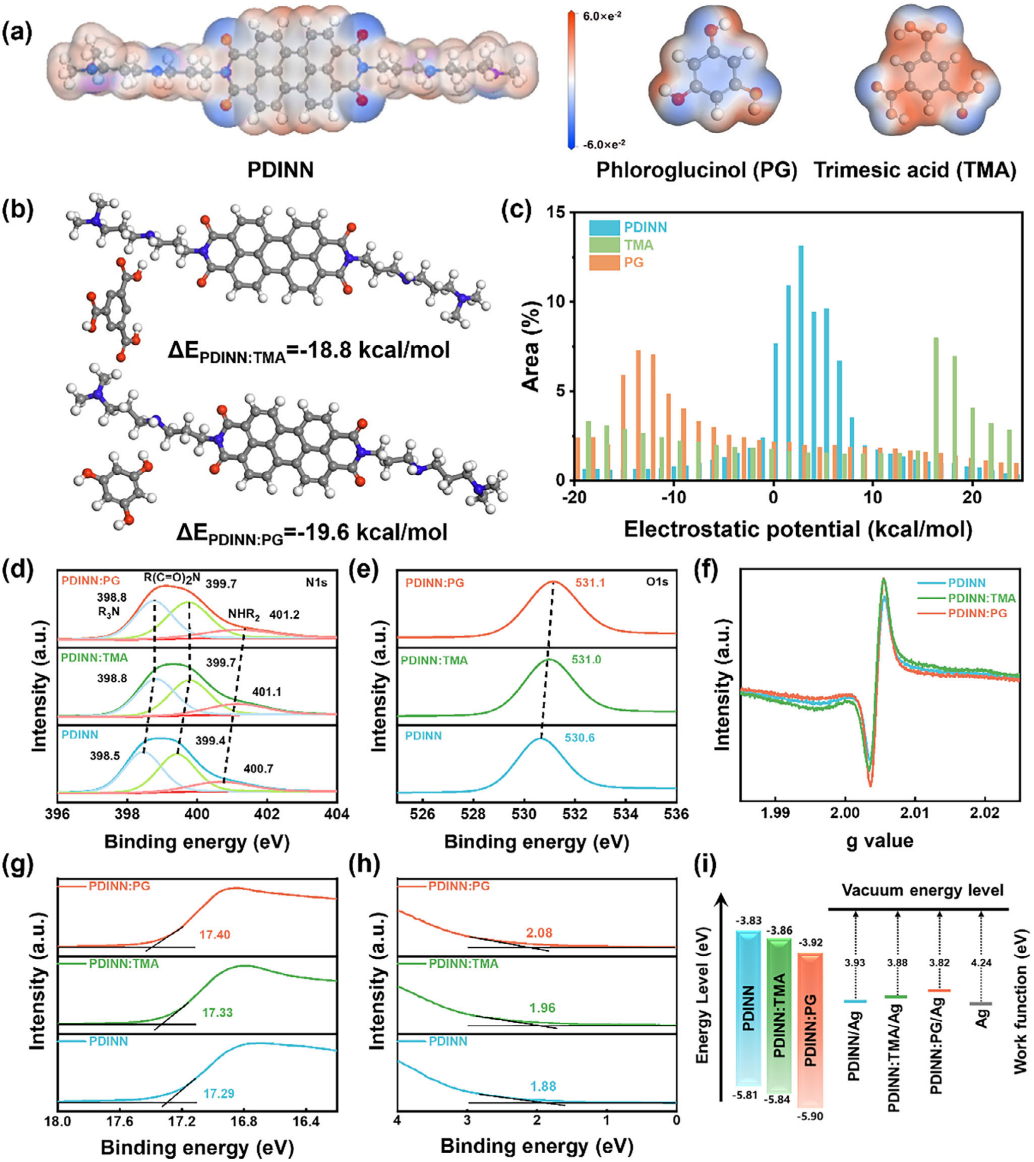
(a) Chemical structures and ESP distributions of PDINN, PG, and TMA. (b) Calculated non‐covalent interaction energies between PDINN and the CIL modifiers (TMA and PG). (c) Quantitative histogram analysis of the ESP area distributions of PDINN, PG, and TMA. (d, e) XPS spectra of (d) N1s and (e) O1s core levels for PDINN, PDINN:TMA, and PDINN:PG. (f) ESR spectra of PDINN, PDINN:TMA, and PDINN:PG films. (g, h) UPS results showing (g) *E_cutoff_* and (h) *E_onset_* of the UPS spectra of PDINN, PDINN:TMA, and PDINN:PG films. (i) Corresponding energy level diagrams of PDINN, PDINN:TMA, and PDINN:PG.

The chemical structures and ESP distributions of PDINN, PG, and TMA were presented in Figure 1a. All three materials exhibited relatively good solubility in alcohol solvents (e.g., methanol) and adequate thermal stability. Thermogravimetric analysis (TGA) revealed 5% weight‐loss temperatures of 205.62°C for PG and 314.34°C for TMA (Figure S2), confirming their suitability for device fabrication. Density functional theory (DFT) calculations illustrated that the molecular surface electrostatic potential of PG was predominantly negative, originating from the electron‐donating conjugative effect of the hydroxyl group. In contrast, the coexistence of weak electron‐donating conjugation and strong electron‐withdrawing inductive effects in the carboxyl group of TMA led to a pronounced separation of positive and negative ESP regions (Figure 1a). To enable quantitative comparison, the average atomic ESP values and ESP area were further analyzed. As illustrated in Figure 1c, PDINN and TMA exhibited predominantly positive ESP distributions, whereas PG displayed a primarily negative ESP distribution. This complementarity is anticipated to strengthen electrostatic interactions between PDINN and PG [31, 32, 33]. Consistently, the calculated intermolecular binding energy of the PG/PDINN complex (−19.6 kcal/mol) was more negative than that of TMA/PDINN (−18.8 kcal/mol), indicating stronger intermolecular interactions in the former system (Figure 1b).

X‐ray photoelectron spectroscopy (XPS) was subsequently employed to probe these interactions experimentally. For pristine PDINN, the N 1s spectrum may be deconvoluted into three components at 398.5, 399.4, and 400.7 eV, corresponding to secondary amine nitrogen, imine nitrogen, and tertiary amine nitrogen species, respectively (Figure 1d). Upon incorporation of either PG or TMA, all N 1s components exhibited systematic shifts toward lower binding energies, indicative of electronic interactions between PDINN and the additives. Meanwhile, the O 1s peak of PDINN appeared at 530.6 eV, whereas those of PDINN:TMA and PDINN:PG shifted to higher binding energies of 531.0 and 531.1 eV, respectively (Figure 1e). Notably, the larger binding energy shifts observed for PDINN:PG relative to PDINN:TMA suggest stronger intermolecular interactions in the PDINN:PG system. These findings were further corroborated by nuclear magnetic resonance (^1^H NMR) spectroscopy (Figure S3). Upon gradual addition of PDINN (50 and 100 µL of PDINN‐dimethyl sulfoxe‐*d*6) to the PG solution, the hydroxyl proton signals of PG exhibited noticeable chemical shifts, confirming their involvement in intermolecular interactions (Figure S3a). More strikingly, the secondary amine proton signal of PDINN shifted downfield from 1.514 ppm to 1.577 and 1.602 ppm upon addition of 50 and 100 µL of PG solution, respectively (Figure S3b), further evidencing a strong interaction between PG and PDINN. Self‐doping effects are known to enhance charge transport in OSCs [34, 35]. To evaluate the self‐doping behavior of the CILs, electron spin resonance (ESR) measurements were performed (Figure 1f). Compared to PDINN and PDINN:TMA, PDINN:PG exhibited a markedly stronger ESR signal with a *g* value of approximately 2.00, arising from unpaired electrons associated with secondary/tertiary amine groups within the polar substituents of the PDI core [36]. This enhanced self‐doping behavior contributes to improved electrical conductivity in CILs [37, 38]. Accordingly, the electrical conductivities of these CIL films were measured using an ITO/CILs/Ag. As shown in Figure S4 and summarized in Table S1, electrical conductivities of 3.94 × 10^−3^, 4.68 × 10^−3^, and 5.67 × 10^−3^ S/cm were obtained for PDINN, PDINN:TMA, and PDINN:PG, respectively, confirming the superior electron‐transport capability of PDINN:PG.

The ultraviolet‐visible (UV–vis) absorption spectra of PDINN, PDINN:TMA, and PDINN:PG films were shown in Figure S5. All three films exhibited nearly identical absorption profiles, with a maximum absorption peak at 476 nm and an absorption onset (*λ_onse_*_t_) at 626 nm. The optical bandgap (*E_g_^op^* ^t^), calculated using the equation $E_{g}^{opt}=1240/\lambda_{onset}$, was 1.98 eV for all three materials. Efficient charge extraction further requires favorable energy‐level alignment between the active layer and CILs. The highest occupied molecular orbital energies (*E_HOMO_*) of PDINN, PDINN:TMA, and PDINN:PG were determined via ultraviolet photoelectron spectroscopy (UPS) using a photon energy of 21.22 eV [39]. Based on the secondary electron cutoff (*E_cutoff_*) and valence band onset (*E_onset_*), the HOMO levels were determined to be −5.81, −5.84, and −5.90 eV, respectively (Table S2). The corresponding lowest unoccupied molecular orbital levels (*E_LUMO_*) were calculated by combining the HOMO energies with the optical band gaps [38], yielding −3.83, −3.86, and −3.92 eV for PDINN, PDINN:TMA, and PDINN:PG, respectively (Table S2). Notably, the LUMO level of PDINN:PG was closer to that of the non‐fullerene acceptor BTP‐eC9 (Figure 1i; Figure S6), facilitating electron extraction. Meanwhile, the deeper HOMO level of PDINN:PG effectively blocked hole transport, thereby suppressing interfacial recombination [40, 41].

Beyond energy‐level alignment, reducing the work function (WF) of the metal electrode is essential for forming Ohmic contacts and minimizing interfacial energy barrier [35, 42, 43]. UPS measurements revealed that deposition of PDINN, PDINN:TMA, and PDINN:PG on Ag induces pronounced shifts in *E_cutoff_* relative to bare Ag (Figure 1g,h). As summarized in Figure 1i, the WF of Ag decreases from 4.24 eV to 3.90, 3.88, and 3.82 eV upon modification with PDINN, PDINN:TMA, and PDINN:PG, respectively, demonstrating the superior WF modulation capability of the additive‐modified CILs. To further elucidate the interfacial interactions responsible for this behavior, XPS analysis of Ag/CIL interfaces was conducted [44]. For Ag/PDINN, the Ag 3d peaks appeared at 367.4 and 373.4 eV. Upon introducing PDINN:TMA, these peaks shifted to 367.7 and 373.7 eV, while further shifts to 367.9 and 373.9 eV were observed for Ag/PDINN:PG (Figure S7a). Concurrently, the N 1s peak shifted from 399.4 eV (Ag/PDINN) to 399.7 eV (Ag/PDINN:TMA) and 399.8 eV (Ag/PDINN:PG) (Figure S7b). The more pronounced binding energy shifts induced by PDINN:PG indicated stronger interfacial interactions, likely arising from enhanced coordination between the amino groups of PDINN and Ag [17, 45]. These interactions promoted a larger interfacial dipolement, leading to a reduced Ag work function (Figure S7c) and improved interfacial contact [46].

**FIGURE 2:**
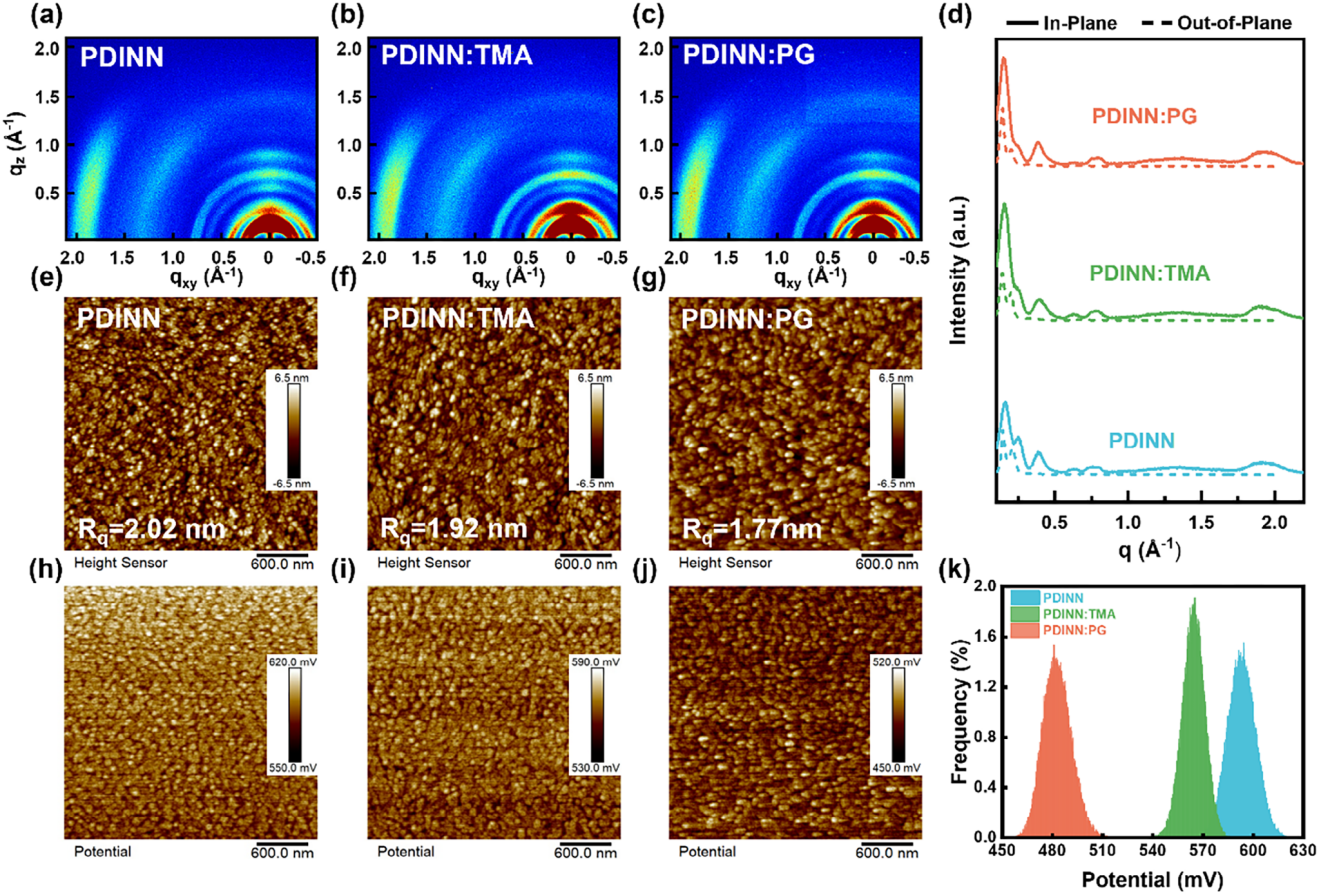
(a–c) GIWAXS pattern of (a) PDINN, (b) PDINN:TMA, and (c) PDINN:PG films. (d) Corresponding line‐cut profiles in the in‐plane (dashed lines) and out‐of‐plane (solid lines) directions. (e–g) AFM height images of (e) PDINN, (f) PDINN:TMA, and (g) PDINN:PG films. (h–j) KPFM surface potential images of (h) PDINN, (i) PDINN:TMA, and (j) PDINN:PG films. (k) Statistical histogram of surface potential distribution for PDINN, PDINN:TMA, and PDINN:PG films.

To investigate the impact of different additives on the crystallinity and molecular packing behavior of PDINN, we conducted grazing incident wide‐angle X‐ray scattering (GIWAXS) measurements [47, 48]. As shown in Figure 2a–c, the GIWAXS patterns of PDINN, PDINN:TMA, and PDINN:PG films exhibited the lamellar stacking (100) peaks in the out‐of‐plane (OOP) direction and *π*–*π* stacking (010) peaks in the in‐plane (IP) direction, suggesting the formation of a preferential edge‐on orientation. The 2D GIWAXS patterns for PDINN, PDINN:TMA, and PDINN:PG were illustrated in Figure 2d. In the IP direction, the *π*–*π* stacking peak positions of PDINN, PDINN:TMA, and PDINN:PG blended films were located at 1.923, 1.928, and 1.937 Å^−1^, corresponding to *d* spacings of 3.27, 3.26, and 3.24 Å, respectively. The crystal coherence lengths (CCLs) of the π–π stacking in the PDINN, PDINN:TMA, and PDINN:PG blended films were calculated to be 22.25, 24.05, and 24.15 Å, respectively (Table S3). This indicated that the introduction of PG materials improved the *π*–*π* stacking of PDINN molecules, and the PDINN:PG films exhibited the highest crystallinity and were expected to achieve better charge transfer [49, 50]. Furthermore, pronounced differences in the lamellar scattering peaks were observed among the pristine PDINN, PDINN:TMA, and PDINN:PG thin films, which were intrinsically correlated with the alkyl chain packing behavior of the conjugated molecules. In the IP direction, the pristine PDINN film displayed multiple lamellar diffraction peaks located at *q*_xy_ = 0.157 Å^−1^ (corresponding crystalline coherence length, CCL = 9.18 nm), 0.250 Å^−1^ (CCL = 7.27 nm), 0.391 Å^−1^ (CCL = 6.42 nm), and 0.765 Å^−1^ (CCL = 5.60 nm). In contrast, the lamellar scattering features of the PDINN: TMA and PDINN:PG blend films were significantly enhanced. Specifically, the diffraction peaks for the PDINN: TMA film were only observed at *q*_xy_ = 0.156 Å^−1^ (CCL = 9.25 nm), 0.397 Å^−1^ (CCL = 5.93 nm), and 0.778 Å^−1^ (CCL = 6.22 nm), whereas the PDINN:PG film showed peaks at *q*_xy_ = 0.156 Å^−1^ (CCL = 9.63 nm), 0.392 Å^−1^ (CCL = 8.34 nm), and 0.783 Å^−1^ (CCL = 6.34 nm). Collectively, these results indicated that PG modification afforded a more pronounced optimization of the lamellar scattering profiles and a greater enhancement of thin‐film crystallinity compared with TMA modification. The improved crystallinity can be primarily attributed to the stronger intermolecular interactions between the hydroxyl moieties of PG and both the oxygen atoms and terminal alkyl moieties of PDINN, which effectively modulated the arrangement and packing mode of the alkyl chains. Such well‐ordered alkyl chain packing contributed to the construction of additional charge‐transport channels, thereby establishing a favorable structural foundation for enhanced charge transport properties in the thin films.

Next, we examined the surface morphology of PDINN, PDINN:TMA, and PDINN:PG films using atomic force microscopy (AFM) and measured the surface potential of the films using Kelvin probe force microscopy (KPFM). As depicted in Figure 2e–g, PDINN, PDINN:TMA, and PDINN:PG films all exhibited similar fibrous structures. The root‐mean‐square roughness (R_q_) values of these films decreased sequentially, from 2.02 nm (PDINN) to 1.92 nm (PDINN:TMA) and further to 1.77 nm (PDINN:PG). Notably, the reduced R_q_ of the PDINN:PG film enabled better ohmic contact with the Ag electrode, which was consistent with the XPS results. The surface potentials of PDINN, PDINN:TMA, and PDINN:PG were measured using KPFM in the Drive Routing to Sample mode, in which a lower surface potential corresponds to a lower work function. As shown in Figure 2h–j, under identical test conditions, the PDINN films exhibited the highest surface potential, followed by PDINN:TMA and PDINN:PG films. This trend was in agreement with the WF trend obtained from UPS measurements. Furthermore, transmission electron microscopy (TEM) measurements were also employed to further investigate the morphologies of the three CILs. In Figure S8a, the PDINN film exhibited obvious aggregation. Although the roughness of the PDINN:TMA film was significantly reduced in the AFM image, inhomogeneous morphological distribution was still observable in the TEM images (Figure S8b). In contrast, the PDINN:PG film showed a distinct fibrous morphology, which is more conducive to the formation of efficient charge transport channels (Figure S8c).

**FIGURE 3:**
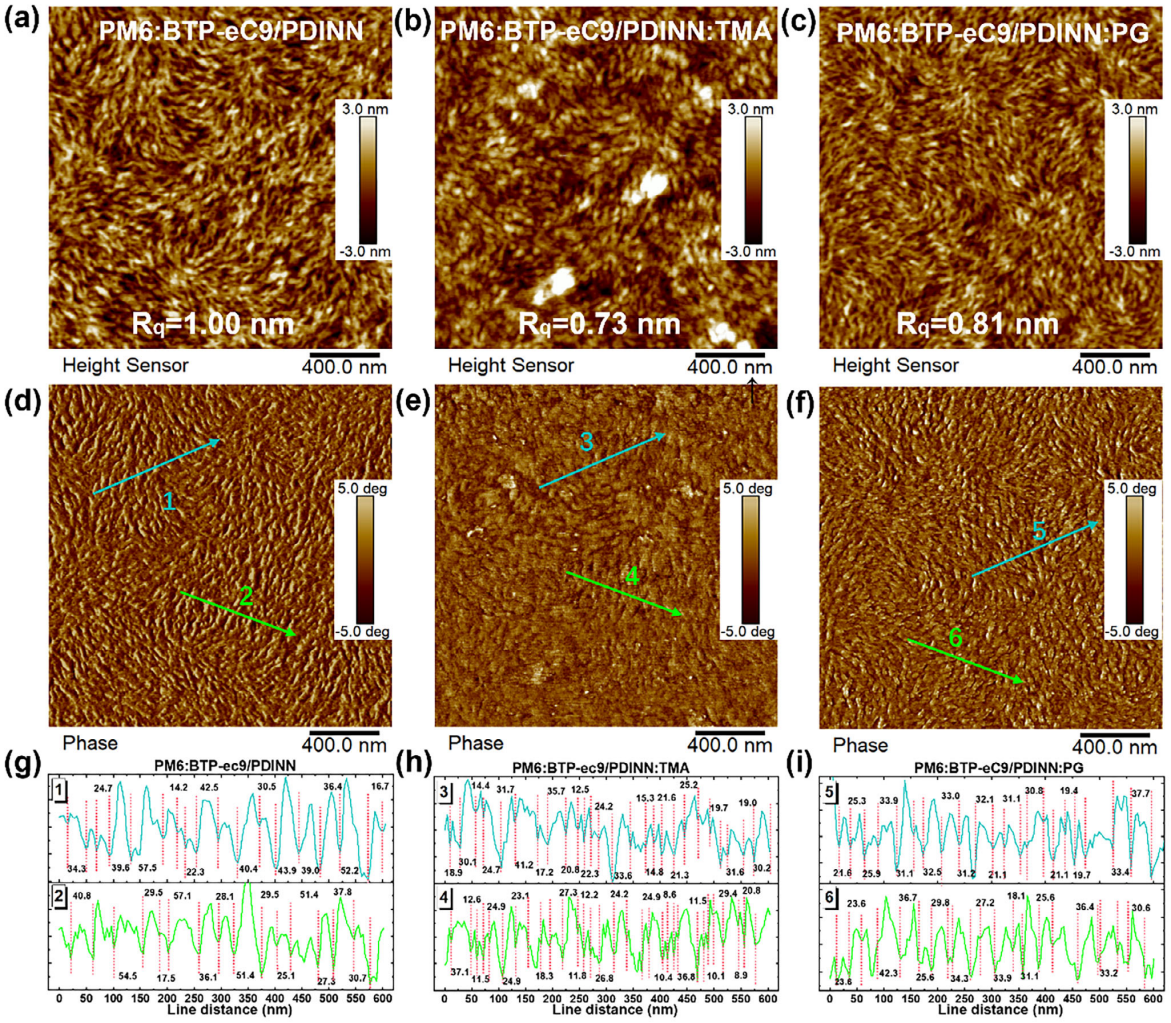
(a–c) AFM height images and (d–f) corresponding phase images of PM6: BTP‐eC9 blend films incorporating PDINN, PDINN:TMA, and PDINN:PG CILs, respectively. (g–i) Statistical analysis of fiber widths extracted from the AFM phase images (defined as the distance between adjacent dashed lines) for PM6: BTP‐eC9/PDINN, PM6: BTP‐eC9/PDINN:TMA, and PM6: BTP‐eC9/PDINN:PG films.

The surface microstructure of the CIL films was also critical in determining the contact at the metal electrode/active layer interface. This aspect was systematically investigated using AFM and surface energy analysis. AFM height images of pristine PM6: BTP‐eC9 and CIL‐modified films (PM6: BTP‐eC9/PDINN, PM6: BTP‐eC9/PDINN:TMA and PM6: BTP‐eC9/PDINN:PG) were shown in Figure 3a–c and Figure S9. All films exhibited characteristic fibrous nanostructures. Notably, deposition of CILs effectively planarizes the initially rough PM6: BTP‐eC9 surface, as evidenced by a reduction in R_q_ from 1.36 nm for the pristine active layer to 1.00 nm with PDINN, 0.81 nm with PDINN:PG, and 0.73 nm with PDINN:TMA. Although PDINN:TMA reduced surface roughness, the interlayer morphology was not fully optimized, likely due to the relatively weaker intermolecular interaction between PDINN and TMA. In contrast, the PDINN:PG modified film exhibited a more uniform and well‐defined fibrous morphology, suggesting the formation of a more ordered interfacial assembly that is favorable for constructing efficient electron‐transport pathways. To further evaluate interfacial compatibility, the surface energies of the active layer and CILs were quantified by measuring the contact angles of water and formamide (Figure S10) [51]. As summarized in Table S4, the surface energies of PM6: BTP‐eC9, PDINN, PDINN:TMA, and PDINN:PG were 11.53, 68.50, 68.30, and 68.24 mN/m, respectively. Importantly, the surface energy of PDINN:PG CIL was closer to that of the active layer, resulting in reduced interfacial energy and improved interfacial compatibility, which facilitated efficient charge transport and collection [52]. Collectively, AFM and contact angle analyses demonstrated that the PDINN:PG CIL established improved interfacial contact with the active layer, thereby promoting more efficient electron extraction and transport.

**FIGURE 4:**
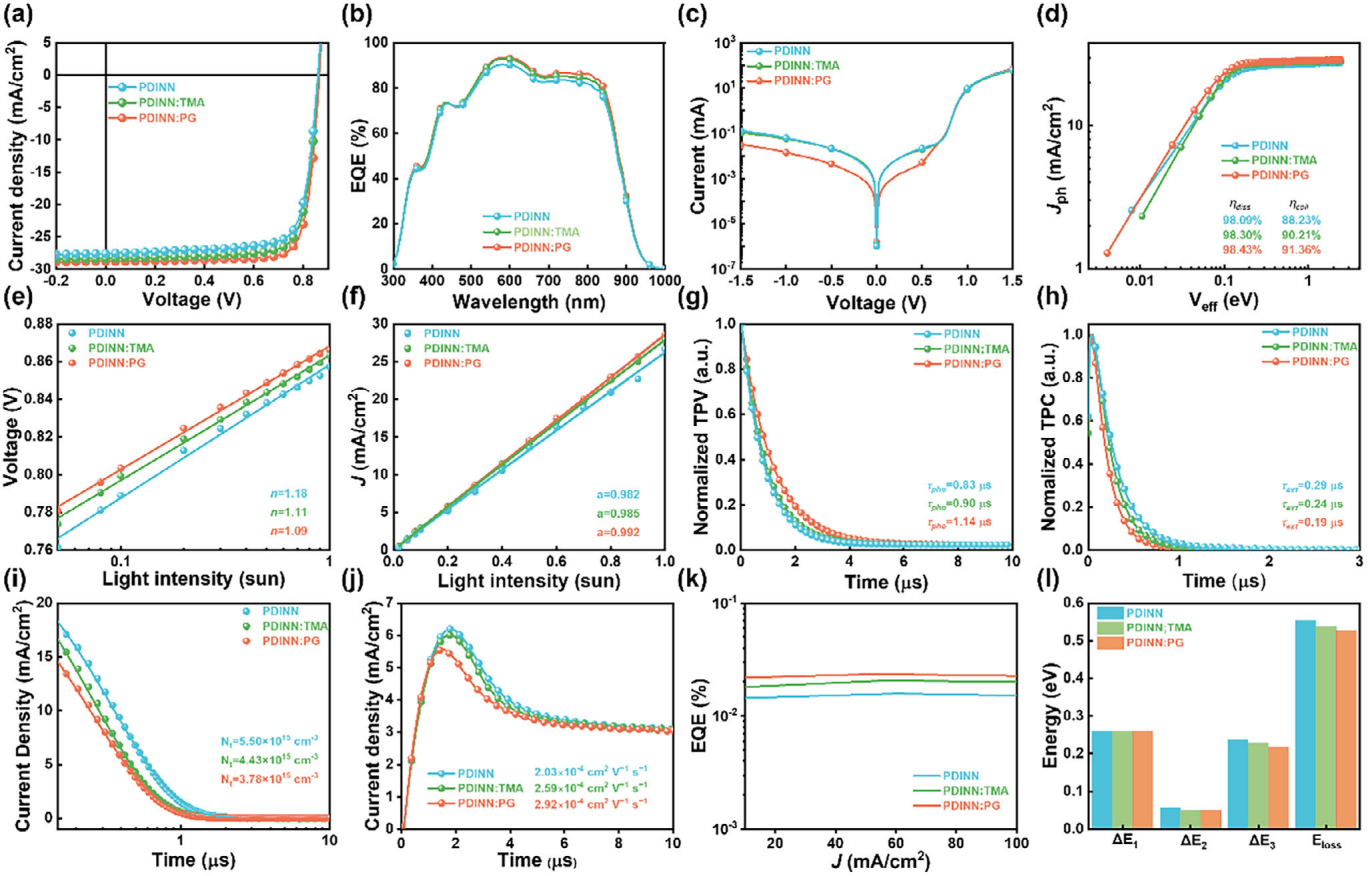
(a) *J*–*V* and (b) EQE curves for PM6: BTP‐eC9 based OSCs containing the PDINN, PDINN:TMA and PDINN:PG CILs. (c) Dark currents of the devices with PDINN, PDINN:TMA, and PDINN:PG CILs. (d) *J_ph_*–*V*_eff_ curve of the devices with PDINN, PDINN:TMA, and PDINN:PG CILs. (e) *V_OC_* and (f) *J_SC_* dependence on the light intensity. (g) TPV and (h) TPC of with PDINN, PDINN:TMA, and PDINN:PG devices. (i) Photo‐CELIV results. (j) The trapped defect state volume density N_t_ of PDINN, PDINN:TMA, and PDINN:PG based devices. (k) The EQE_EL_ spectra, and (l) column plots of energy loss breakdown analysis of the OSCs based on PDINN, PDINN:TMA, and PDINN:PG CILs.

To systematically evaluate and compare the photovoltaic performance of devices incorporating different CILs, OSCs were fabricated using a conventional device configuration of ITO/2Br‐2PACz/PM6: BTP‐eC9/CIL/Ag, as illustrated in Figure S11a. The concentrations of PG and TMA were carefully optimized, with the corresponding results summarized in Figure S11b,c and Table S5. The control devices based on pristine PDINN exhibited an open‐circuit voltage (*V_OC_*) of 0.857 V, a short‐circuit current density (*J_SC_*) of 27.58 mA/cm^2^, and a fill factor (FF) of 77.4%, yielding a PCE of 18.3%. By contrast, devices incorporating PDINN:TMA delivered improved performance, with a *V_OC_* of 0.858 V, a *J_SC_* of 28.33 mA/cm^2^, and an FF of 78.7%, resulting in an improved PCE of 19.1%. For PDINN:PG‐based devices, the optimal concentration of PG was determined to be 1.0 mg/mL (Figure 4a and Table 1). The champion PDINN:PG devices achieved a higher PCE of 20.0%, outperforming both PDINN (18.3%) and PDINN:TMA‐based (19.1%) devices, owing to simultaneous improvements in all three key photovoltaic parameters (*V_OC_* = 0.863 V, *J_SC_* = 28.82 mA/cm^2^, FF = 80.6%). Notably, a certified efficiency of 19.5% was independently validated by the South China National Center of Metrology (Guangdong Institute of Metrology, Figure S13). These photovoltaic metrics rank among the top‐tier values reported for binary OSCs. Moreover, PDINN:PG‐based devices exhibited the highest average PCE of 19.9%, surpassing those of PDINN (18.0%) and PDINN:TMA (18.9%) devices, based on statistical analysis of at least 10 independent devices (Table 1; Figure S12). Importantly, the discrepancy between the *J_SC_^a^* values obtained from *J*–*V* measurements and those integrated from the external quantum efficiency (EQE) curve was below 5%, confirming the reliability of the data (Table 1). As shown in Figure 4b, devices incorporating PDINN:TMA and PDINN:PG exhibited enhanced EQE responses mainly in the long‐wavelength region from 520 to 850 nm, indicative of improved photon‐to‐electron conversion efficiency.

**TABLE 1:** The photovoltaic parameters of PM6: BTP‐eC9 based OSCs with PDINN, PDINN:TMA, and PDINN:PG CILs under the AM 1.5G (100 mW/cm^2^) light source illumination.

CIL	*V_OC_* (V)	*J_SC_* (mA/cm^2^)	FF (%)	PCE (%)	*J_SC_^a^*
PDINN	0.857 (0.856 ± 0.002)	27.58 (27.22 ± 0.18)	77.4 (77.1 ± 0.4)	18.3 (18.0 ± 0.2)	26.55
PDINN:TMA	0.858 (0.858 ± 0.001)	28.33 (28.06 ± 0.15)	78.7 (78.5 ± 0.2)	19.1 (18.9 ± 0.1)	27.20
PDINN:PG	0.863 (0.863 ± 0.001)	28.82 (28.69 ± 0.11)	80.6 (80.2 ± 0.3)	20.0 (19.9 ± 0.1)	27.55

^a)^ Integrated *J_SC_^a^* from the EQE curves.

^b)^ The average parameters of PM6: BTP‐eC9 based OSCs were calculated across 10 ndependent cells.

To elucidate the influence of different CILs on carrier transport and recombination, dark *J*–*V* characteristics were measured (Figure 4c). Compared with PDINN and PDINN:TMA‐based devices, the PDINN:PG device exhibited a significantly reduced leakage current and an enhanced rectification ratio, indicative of improved charge selectivity and suppressed charge recombination, consistent with its higher *J_SC_* and FF. Charge extraction behavior was further investigated by analyzing the relationship between the photocurrent density (*J_ph_*) and the effective voltage (*V_eff_*) of the devices [50, 53, 54], as shown in Figure 4d. Here, *J_ph_* is defined as *J_ph_* = *J_L_*  − *J_D_*, where *J_L_* and *J_D_* denoted the photocurrent density under light and dark conditions, respectively. *V_eff_* is calculated as *V_eff_* = *V*_0_  − *V_a_*, where *V_0_* is the voltage when *J_ph_* = 0 and V_a_ is the applied bias [49]. When V_eff_ reaches 2 V, photogenerated excitons dissociate completely, resulting in the saturated photocurrent density (*J_sat_*). The exciton dissociation efficiency (η_*diss*_ = *J_ph_* /*J_sat_*.) was calculated to be 98.09%, 98.22% and 98.43% for PDINN, PDINN:TMA, and PDINN:PG devices, respectively (Table S6). The charge collection efficiency (η_*coll*_ = *J*_max *power*_ /*J_sat_*) [55] of the PDINN:PG device reached 91.36%, exceeding those of the PDINN:TMA (90.21%) and PDINN(88.23%) devices, indicating more efficient charge extraction and collection. To evaluate the interfacial resistance in the OSCs, electrochemical impedance spectroscopy (EIS) was employed, and the resulting Nyquist plots were fitted using an equivalent circuit model [56, 57]. As shown in Figure S14, devices incorporating three different CILs exhibited a decreasing trend in series resistance (R_s_), following the order: R_s_ (PDINN:PG) < R_s_ (PDINN: TMA) < R_s_ (PDINN). The reduced R_s_ was attributed to the improved interfacial contact established by the PDINN:PG layer between the active layer and the cathode, which facilitated more efficient charge transport and extraction at the electrode interface. This result was consistent with the higher *J_SC_* and FF achieved by the PDINN:PG‐based devices, as discussed below.

Charge recombination kinetics were further analyzed by examining the dependence of *J_SC_* and Voc on light intensity (*P_light_*), following the relationships *V_OC_* ∝ *n*(*kT*/*q*)ln(*P_light_*) and *J_SC_* ∝ *P_light_^α^*, where an α value close to 1 indicates the suppression of bimolecular recombination, and an *n* value close to 1 indicates minimal trap‐assisted recombination [43, 58]. As shown in Figure 4e,f, the PDINN:PG device (α = 0.992, *n* = 1.09) was closer to the ideal values than the PDINN (α = 0.982, *n* = 1.18) and PDINN:TMA (α = 0.985, *n* = 1.11), suggesting that both bimolecular and trap‐assisted recombination were effectively suppressed. Transient photocurrent (TPC) and transient photovoltage (TPV) measurements were subsequently performed to quantitatively probe charge extraction and recombination dynamics [59, 60, 61, 62]. As shown in Figure 4g, the charge extraction times (*τ_ext_*) of the PDINN:PG device (0.19 µs) were significantly shorter than those of the PDINN device (0.29 µs), indicating faster charge extraction. TPV measurements under open‐circuit voltage conditions revealed the carrier lifetime (*τ_pho_*) of 0.83, 0.90, and 1.14 µs for PDINN, PDINN:TMA, and PDINN:PG devices, respectively (Figure 4h). The combination of faster charge extraction and longer carrier lifetime accounts well for the enhanced *J_SC_* and FF of the PDINN:PG device.

Charge‐carrier transport properties were further investigated using the space‐charge‐limited current (SCLC) method. As depicted in Figure S15, the electron mobilities (µ_e_) of PDINN, PDINN:TMA, and PDINN:PG devices were determined to be 3.22 × 10^−4^, 5.21 × 10^−4^, and 6.70 × 10^−4^ cm^2^ V^−1^ s^−1^, respectively. In addition, photo‐induced charge carrier extraction by linearly increasing voltage (Photo‐CELIV) measurements were performed to probe the charge carrier generation and transport under operational conditions [48]. As presented in Figure 4i, the extracted carrier mobilities were 2.03 × 10^−4^, 2.59 × 10^−4^, and 2.92 × 10^−4^ cm^2^ V^−1^ s^−1^ for PDINN, PDINN:TMA, and PDINN:PG devices, respectively, further confirming the superior charge transport capability of PDINN:PG‐based devices. Trap‐state densities were evaluated from the trap‐filled limit voltage (*V_TFL_*) using the equation: *N*_trap_ =  2ε0εr*V_TFL_*/*qL* ^2^ [63]. As summarized in Figure S15b–d and Table S7, the trap densities decreased from 2.87 × 10^18^ cm^−3^ (PDINN) to 2.68 × 10^18^ cm^−3^ (TMA) and 2.36 × 10^18^ cm^−3^ (PDINN:PG). Furthermore, current‐based Deep‐Level Transient Spectroscopy (DLTS) measurements under a reverse bias of −3.0 V revealed that the PDINN:PG device exhibited the lowest trapped defect state volume density (N_t_ = 3.78 × 10^15^ cm^−3^), compared with 4.43 × 10^15^ cm^−3^ for PDINN:TMA and 5.50 × 10^15^ cm^−3^ for PDINN (Figure 4j). These results indicate that PG effectively improves interfacial contact, suppresses defect‐assisted recombination, and enhances photovoltaic performance.

To further elucidate the origin of the improved *V_OC_*, energy loss (E_loss_) analysis was conducted based on Shockley‐Queisser (SQ) theory, where E_loss_ was divided into three components: ΔE_1_ (unavoidable radiative loss), ΔE_2_ (sub‐bandgap radiative loss), and ΔE_3_ (non‐radiative loss) [64, 65]. The corresponding results were presented in Figure 4l and Table S8. Owing to similar optical band gaps, all devices exhibited comparable ΔE_1_ values of 0.26 eV. The ΔE_2_ values for PDINN:PG and PDINN:TMA devices were both 0.051 eV, slightly lower than that of the PDINN device (0.057 eV), reflecting reduced sub‐bandgap radiative recombination. The Urbach energy (E_u_), extracted from Fourier transform photocurrent spectroscopy‐external quantum efficiency (FTPS‐EQE), provided further insight into energy disorder. As shown in Figure S16 and Table S8, the E_u_ values of the PDINN:PG (18.64 meV) and PDINN:TMA (19.21 meV) devices were lower than that of the PDINN device (19.76 meV), indicating improved molecular ordering and reduced energy disorder upon additive incorporation. The non‐radiative energy loss ΔE_3_, calculated from ΔE_3_ = −kT(lnEQE_EL_), decreased from 0.237 eV (PDINN) to 0.229 eV (PDINN:TMA) and further to 0.217 eV (PDINN:PG), corresponding to enhanced EQE_EL_ values of 0.011%, 0.015%, and 0.023%, respectively (Figure 4l). Consequently, the total energy loss of the PDINN:PG device was reduced from 0.554 to 0.528 eV, leading to a pronounced improvement in *V_OC_*.

**FIGURE 5:**
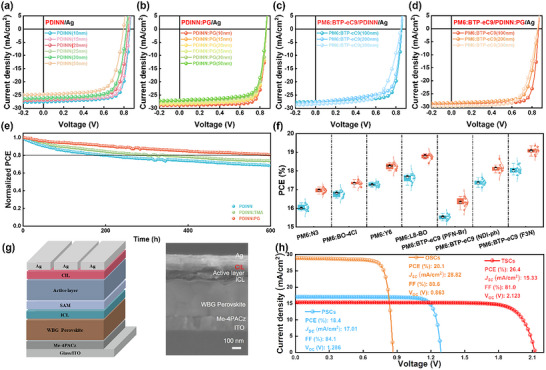
*J*–*V* curves of the OSCs based on PM6: BTP‐eC9 with (a) PDINN and (b) PDINN:PG CILs of different thicknesses. *J*–*V* curves of OSCs with PM6: BTP‐eC9 active layers of different thicknesses, using (c) PDINN and (d) PDINN:PG as the CILs. (e) The extrapolating MPP curves of the devices with PDINN, PDINN:TMA, and PDINN:PG. (f) Statistics of PCE in various systems (different donors and acceptors) averaged by at least 10 devices. The black and red data are PDINN and PDINN:PG, respectively. (g) Schematic and cross‐sectional SEM image of the TSCs device structure; (h) *J*–*V* curves of OSCs, PSCs, and TSCs.

The thickness of CILs is typically constrained to below 10 nm, which imposes significant challenges for device fabrication, particularly in large‐area processing. Recognizing the critical role of CIL film thickness sensitivity in practical device fabrication, we systematically investigated the photovoltaic performance of devices with varying CIL thicknesses. All photovoltaic parameters were obtained from statistical analysis of at least 10 independent devices (Table S9). As shown in Figure 5b and Table S9, when PDINN:PG was employed as the CIL, the devices exhibit remarkable tolerance to film thickness variations. Within a thickness range of up to 30 nm, the average PCE of PDINN:PG‐based devices remained as high as 89.5% of the optimal value. Even at a thickness of 50 nm, the devices still retained 87.0% of the optimal PCE, demonstrating excellent robustness. This outstanding thickness tolerance can be attributed to the stronger self‐doping characteristics of PDINN:PG, which endows it with superior electron transport capability and enables efficient charge extraction even at relatively large film thicknesses. Moreover, compared with the control devices (Figure 5c,d), PDINN:PG devices also exhibited exceptional tolerance toward thicker active layers. When the active layer thicknesses increased to 200 and 300 nm, the control devices achieved PCEs of 16.8% and 15.7%, respectively, whereas the corresponding PDINN:PG‐based devices maintained high PCEs of 18.2% and 17.6% (Table S10), further underscoring the robustness of this interfacial engineering strategy.

Device stability, a key factor for the commercial viability of OSCs, was subsequently evaluated (Figure 5e). To accurately assess operational stability, the as‐fabricated devices were stored in a nitrogen atmosphere and subjected to maximum power point (MPP) tracking measurements under 100 mW/cm^2^ white LED illumination using a LED solar simulator (SLS‐LED‐80). The PDINN:PG devices demonstrated markedly improved photostability, maintaining 80.6% of their initial PCE after 600 h of operation, outperforming the PDINN:TMA‐based (73.7%) and PDINN‐based (68.8%) devices. This enhanced stability is primarily attributed to suppressed trap‐assisted recombination and improved interfacial compatibility between the CIL and the active layer. To further examine the universality of the hybrid CIL strategy incorporating PDINN:PG, a series of benchmark active layers, including PM6: N3, PM6: BO‐4Cl, PM6: Y6, and PM6: L8‐BO (structures shown in Figure S17a), were employed. As summarized in Figure 5d and Table S11, hybrid CILs containing PDINN:PG consistently enhanced device performance across all systems, demonstrating excellent universality. To further validate the general applicability of the PG‐based molecular interfacial engineering strategy, three additional CIL materials (PFN‐Br, NDI‐ph, and PNDIT‐F3N, structures shown in Figure S18a) were incorporated into PM6: BTP‐eC9‐based OSCs. As illustrated in Figure S18b and Table S12, the introduction of PG into PDIN, PDINO, and F3N produces a positive regulatory effect analogous to that of the PDINN‐based CIL system, leading to significantly enhanced PCEs compared to their respective pristine counterparts. All photovoltaic parameters were obtained from statistical analysis of at least 10 independent devices (Figure 5f). These results confirm the broad versatility of PG across diverse CIL platforms. Encouraged by the excellent universality of this molecular interfacial engineering strategy, we further extended it to the fabrication of perovskite‐organic tandem solar cells. Figure 5g presents the device structure architecture of the perovskite‐organic TSCs with the configuration: ITO/Me‐4PACz/WBG perovskite/ICL/SAM/PM6: BTP‐eC9/PDINN:PG/Ag. The corresponding *J*–*V* characteristics of the OSCs, single‐junction perovskite solar cells (PSCs), and tandem devices were presented in Figure 5h. The optimal TSCs device exhibited an outstanding PCE of 26.4%, with a high *V_OC_* of 2.123 V, a *J_SC_* of 15.33 mA/cm^2^, and an impressive FF of 81.0%. As shown in Figure S19, the perovskite‐organic TSCs showed excellent current‐matching characteristics and complementary light absorption across a broadened spectral range, where the *J_SC_* values of 14.61 and 14.22 mA/cm^2^ are integrated from the EQE spectra of the PSCs and OSCs subcells, respectively. These results further substantiate the effectiveness and universality of the proposed molecular interfacial engineering strategy for high‐efficiency photovoltaic devices.

## 3 Conclusions

In summary, we report a high‐performance hybrid CIL, PDINN:PG, realized by incorporating a multifunctional PG molecule into PDINN. Combined theoretical and experimental analyses reveal that electrostatic potential complementarity and strengthened intermolecular interactions synergistically regulate the physicochemical properties of the interlayer. As a result, PDINN:PG CIL exhibits enhanced self‐doping characteristics, improved electrical conductivity, optimized energy‐level alignment, reinforced molecular packing with stronger *π*–*π* interactions, and an effectively reduced Ag electrode WF. These collective effects enable more efficient charge extraction and transport while substantially suppressing bimolecular and trap‐assisted recombination, thereby minimizing non‐radiative energy losses. Consequently, OSCs employing PDINN:PG achieve a champion PCE of 20.0% (certified 19.5%), outperforming control devices based on pristine PDINN (18.3%) or PDINN:TMA (19.1%). Notably, the simultaneous enhancement of *V_OC_*, *J_SC_*, and FF underscores the effectiveness of the interfacial modulation strategy. Beyond efficiency gains, PDINN:PG endows devices with exceptional tolerance to interlayer and active‐layer thickness variation, improved operational stability, and broad compatibility with diverse photoactive systems, extending its applicability to high‐efficiency perovskite‐organic TSCs. Overall, this work provides a practical strategy and fundamental design principles for constructing high‐performance, thickness‐tolerant, and durable cathode interlayers through molecular interfacial engineering.

## Author Contributions

Conceptualization, X.D., J.L., A.N., H.Z., and H.H.; Methodology and critical data curation, X.D., J.L.; Data curation – D.M., J.C., G.Z., X.H., H.L., P.F., Z.R., G.L., and M.Y.; Writing – original draft, X.D.; Writing – review & editing, J.L., A.N., H.Z., and H.H.; Supervision, J.L., A.N., H.Z., and H.H.

## Conflicts of Interest

The authors declare no conflicts of interest.

## Supporting information




**Supporting File**: advs75134‐sup‐0001‐SuppMat.docx.

## Data Availability

The data that support the findings of this study are available from the corresponding author upon reasonable request.
