# Supplementary material for: Electrostatic Potential Complementarity for Thickness‐Tolerant Cathode Interlayers in High‐Efficiency Organic and Tandem Solar Cells
Source: Adv Sci (Weinh). 2026 Apr 2;13(36):e75134. doi: 10.1002/advs.75134 (PMC13317574; doi:10.1002/advs.75134)
Supplement: Supplementary file 1 — Supporting File: advs75134‐sup‐0001‐SuppMat.docx. [file ADVS-13-e75134-s001.docx]

Supporting Information

DOI: 10.1002/((please add manuscript number))

**Article type: research articles**

**Electrostatic Potential Complementarity for Thickness-Tolerant Cathode Interlayers in High-Efficiency Organic and Tandem Solar Cells**

Xiaoman Ding,^1,2^ Dana Mukasheva,^3^ Jiaxu Che,^4^ Guangye Zhang,^4^ Xiuze Hei,*^1^ Haoran Lin,^1^ Jie Lv,*^1^ Patrick Fong,^5^ Zhiwei Ren,^5^ Annie Ng,*^3^ Mingjian Yuan,^6^ Hongyu Zhang,*^2^ Gang Li,^5^ Hanlin Hu*^1^

^1^X. Ding, Prof. X. Hei, Prof. H. Lin, J. Lv, Prof. H. Hu

Hoffmann Institute of Advanced Materials, Shenzhen Polytechnic University, 7098 Liuxian Boulevard, Shenzhen 518055, China.

E-mail: [xiuzehei@szpu.edu.cn](mailto:xiuzehei@szpu.edu.cn)

E-mail: jie-hanm.lyu@polyu.edu.hk

E-mail: hanlinhu@szpu.edu.cn

^2^X. Ding, Prof. H. Zhang,

State Key Laboratory of Supramolecular Structure and Materials, College of Chemistry, Jilin University, Changchun, 130012, China.

E-mail: hongyuzhang@jlu.edu.cn

^3^Dana Mukasheva, Prof. Annie Ng

Department of Electrical and Computer Engineering, School of Engineering and Digital Sciences, Nazarbayev University, 53 Kabanbay Batyr Avenue, Astana, 010000, Kazakhstan.

E-mail: annie.ng@nu.edu.kz

^4^J. Che, Prof. G. Zhang

College of New Materials and New Energies, Shenzhen Technology University, Shenzhen, 518118, China.

^5^Dr. P. Fong, Prof. Z. Ren, Prof. G. Li

Department of Electrical and Electronic Engineering, Research Institute for Smart Energy (RISE), Photonic Research Institute (PRI), The Hong Kong Polytechnic University, Hung Hom, Kowloon, Hong Kong SAR 999077, China.

^6^Prof. M. Yuan

College of Chemistry, Nankai University, Tianjin, 300071, China.

**Content**

[1. Device Fabrication 5](#_Toc27012)

[2. Current Density-Voltage (](#_Toc5892)*[J-V](#_Toc5892)*[) and External Quantum Efficiency (EQE) Measurements 8](#_Toc5892)

[3. The thermal decomposition temperature of Materials 10](#_Toc25281)

[4. Density Functional Theory (DFT) 11](#_Toc8004)

[5.](#_Toc30008) ^[1](#_Toc30008)^[H NMR spectrum of Materials 12](#_Toc30008)

[6. Conductivity of CILs 13](#_Toc20688)

[7. UV-vis absorption spectra of Materials 14](#_Toc26080)

[8. Energy level of Materials 15](#_Toc11926)

[9. X-ray photoelectron spectroscopy (XPS) Measurements 17](#_Toc32373)

[10. Grazing Incidence Wide-angle X-ray Scattering (GIWAXS) 18](#_Toc3010)

[11. Transmission Electron Microscopy (TEM) and Element Mapping Diagrams 20](#_Toc15438)

[12. Atomic Force Microscopy (AFM) and Kelvin Probe Force Microscopy (KPFM) 21](#_Toc29634)

[13. Contact Angle Measurements 22](#_Toc15047)

[14. OSCs Device Performance Data 24](#_Toc903)

[15. Exciton dissociation data 29](#_Toc24078)

[16. Electrochemical Impedance Spectroscopy (EIS) 30](#_Toc17813)

[17. SCLC Mobility Measurements and Trap Density 31](#_Toc10586)

[18. Energy Losses (E](#_Toc28649)_[loss](#_Toc28649)_[) 33](#_Toc28649)

[19. Photovoltaic Performance Data of OSCs 35](#_Toc17364)

[20. The Maximum Power Point (MPP) tracking Measurements 40](#_Toc6590)

[21. The External Quantum Efficiency (EQE) date of TSCs 41](#_Toc2482)

[22. References 42](#_Toc28846)

**1. Device Fabrication**

The PM6: BTP-eC9 organic solar cells were prepared on glass substrates with tin-doped indium oxide (ITO, 15 Ω/sq) patterned on the surface (device area: 0.1 cm^2^). Substrates were prewashed with isopropanol to remove organic residues before immersing in an ultrasonic bath of soap for 15 min. Samples were rinsed in flowing deionized water for 5 min before being sonicated for 15 min each in successive baths of deionized water, acetone and isopropanol. Next, the samples were dried with pressurized nitrogen before being exposed to a UV-ozone plasma for 15 min. The PEDOT: PSS and 2Br-2PACz were selected as a hole transport layer, respectively. 2Br-2PACz was dissolved in isopropanol solution (0.3 mg/mL), a thin layer was spin-coated onto the UV-treated substrates, the substrates were subsequently annealed on a hot plate at 100 °C for 10 min, and PEDOT: PSS was spin-coated onto the UV-treated substrates, the substrates were subsequently annealed on a hot plate at 150 °C for 10 min, and the substrates were then transferred into the glovebox for active layer deposition. All solutions were prepared in the glovebox using the donors of PM6, the acceptors of BTP-eC9. The PEDOT: PSS was purchased Xi'an Yuri Solar Co,. Ltd., the PM6 was purchased Volt-Amp Optoelectronics Tech. Co., Ltd, Dongguan, China; 2Br-2PACZ, BTP-eC9, and PDINN were purchased from Organtec. Ltd, and the ITO glass was purchased from Advanced Election Technology Co. Ltd. The TMA and PG were purchased from TCI and the ITO glass was purchased from Advanced Election Technology Co. Ltd. The devices were obtained by dissolving PM6 and BTP-eC9 in CF using a D/A ratio of 1: 1.2, and a total concentration of 15.4 mg/mL. The active layer dissolved in CF was spun coated at a speed of 3500 rpm and for 30 s, respectively, resulting in films of 95 to 105 nm in thickness. The active layer was thermal annealing (TA) for 5 min at 80 °C. The next stage is to coat CILs on active layer, about 40 µL PDINN, PDINN:TMA and PDINN:PG solution (2 mg/mL in Methanol) was spin-coated at 2500 rpm for 20s. Optimized devices were obtained by dissolving TMA and PG in PDINN solution at concentrations of 0.05 mg/mL, 0.10 mg/mL and 0.15 mg/mL, respectively. Finally, the samples were placed in a thermal evaporator for evaporation of a 100 nm-thick layer of Silver (Ag) evaporated at 2 Å/s; pressure of less than 2x10^-6^ Torr.

**Wide-bandgap perovskite single-junction device fabrication**

The substrates were then spin-coated with NiO_X_ (10 mg/mL in deionized water) nanoparticle dispersion. Then, the films were transferred to a nitrogen-filled glovebox and the Me-4PACz (1 mg/mL in IPA) was spin-coated on the substrates at 4,000 rpm for 30 s and heated at 100℃ for 10 min. The 1 M perovskite (Cs_0.25_FA_0.75_Pb(Br_0.5_I_0.5_)_3_) precursor solution used contains 129 mg FAI, 65 mg CsI, 275 mg PbBr_2_, and 115 mg PbI_2_, which were dissolved in 1 mL mixed solvent of DMF: DMSO (v/v = 4:1). Then, the precursor solution was shaken overnight at 60℃. For the spin-coating process, the substrate was spun at 4,000 rpm for 45 s with an acceleration of 4,000 rpm/s, and 200 mL of MeAc was slowly dropped at 15 s before the spin-coating ended. The perovskite

films were then annealed at 100℃ for 15 min. Then, the C60 (10 nm)/BCP (5 nm)/Ag (100 nm) layers were deposited to complete the device fabrication.

**Perovskite-organic tandem device fabrication**

After completing the deposition of the BCP layer, Ag (1 nm)/MoO_X_ (15 nm) was thermally evaporated on top of BCP and the film was brought back to the nitrogen-filled glovebox, then completed the deposition of the SAM layer. For the narrow band-gap PM6: BTP-eC9 system, the solution was prepared by dissolving blends with a weight ratio of 1:1.2 in chloroform (total concentration 15.4 mg/mL) and stirred at room temperature for 2 hours. The active layer was spin-coated on the ITO/SAM substrates at 3500 rpm and then annealed 5 min at 80 ℃. Finally, PDINN:PG solution (2 mg/mL in Methanol) was spin-coated at 2500 rpm for 20s.

**2. Current Density-Voltage (*J-V*) and External Quantum Efficiency (EQE) Measurements**

The *J*-V curves of devices were measured using a Keithley 2400 Source Meter in glove box under AM 1.5G (100 mW/cm^2^) using a Enlitech solar simulator (purchased from Enli Technology Co., Ltd.). A 2×2 cm^2^ monocrystalline silicon reference cell with KG1 filter (purchased from Enli Tech. Co., Ltd., Taiwan). During the *J-V* curves testing, a four-wire connection was employed, and the device was covered with the Mask (0.0804 cm^2^) depicted in **Figure S1a** to ensure accurate area measurement. The device area is 0.1 cm^2^.


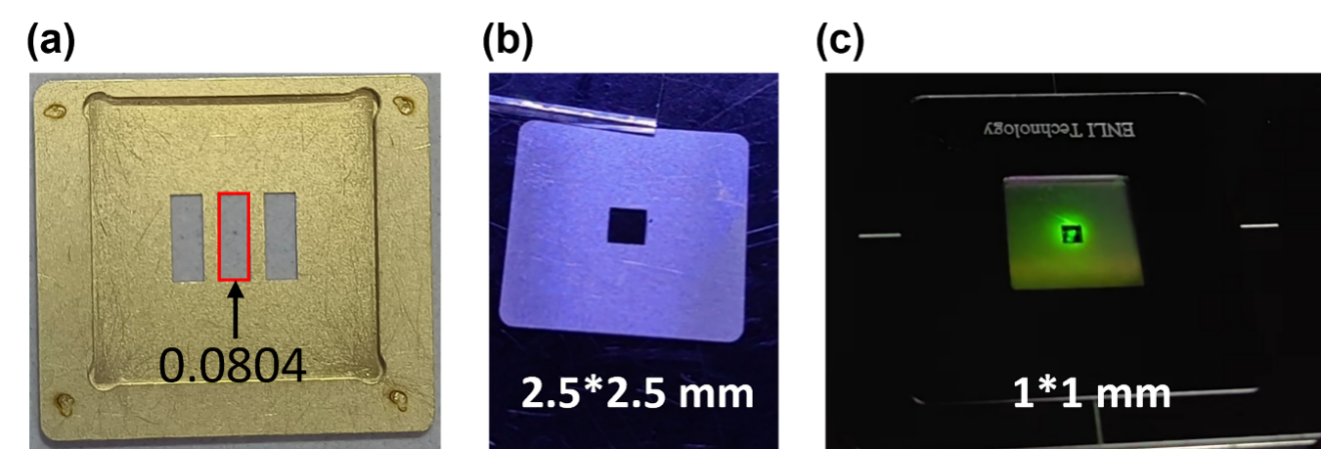


**Figure S1.** (a) The mask used during *J-V* testing has an area of 0.0804 cm^2^; (b) Square hole mask used for calibration of silicon batteries in EQE, with an area of 2.5×2.5 mm^2^; (c) The size of the spot used for EQE testing, with an area of 1×1 mm^2^.

The EQE was measured by a certified incident photon to electron conversion (IPCE) equipment (QE-R) from Enli Technology Co., Lt. The light intensity at each wavelength was calibrated using a standard monocrystalline Si photovoltaic cell. As shown in **Figure S1b**, the photovoltaic cell was covered with a mask template featuring holes measuring 2.5×2.5 mm^2^ to ensure precise alignment of a 1×1 mm^2^ EQE spot in this region. The 1×1 mm^2^ spot is then directed onto the effective area of the device under test for EQE testing.

**3. The thermal decomposition temperature of Materials**


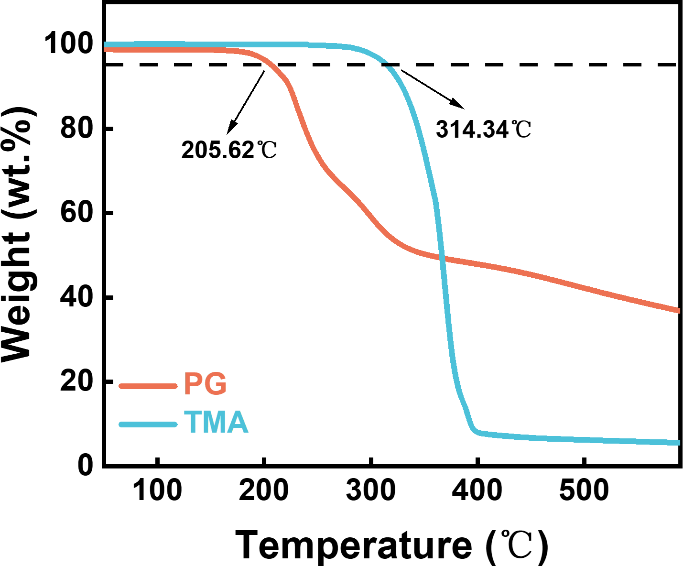


**Figure S2.** TGA curves of PG and TMA.

**4. Density Functional Theory (DFT)**

All the calculations of the model compounds studied in this work were performed using the Gaussian 09 software package. Ground state geometry optimizations of PDINN, TMA and PG are calculated by DFT at the B3LYP/6-31G (d, p) level. Then the ESP characteristic parameters were calculated based on the optimal structure with electronic wave function information using Multiwfn 3.7(dev) program.^[1]^

**5. ^1^H NMR spectrum of Materials**

**
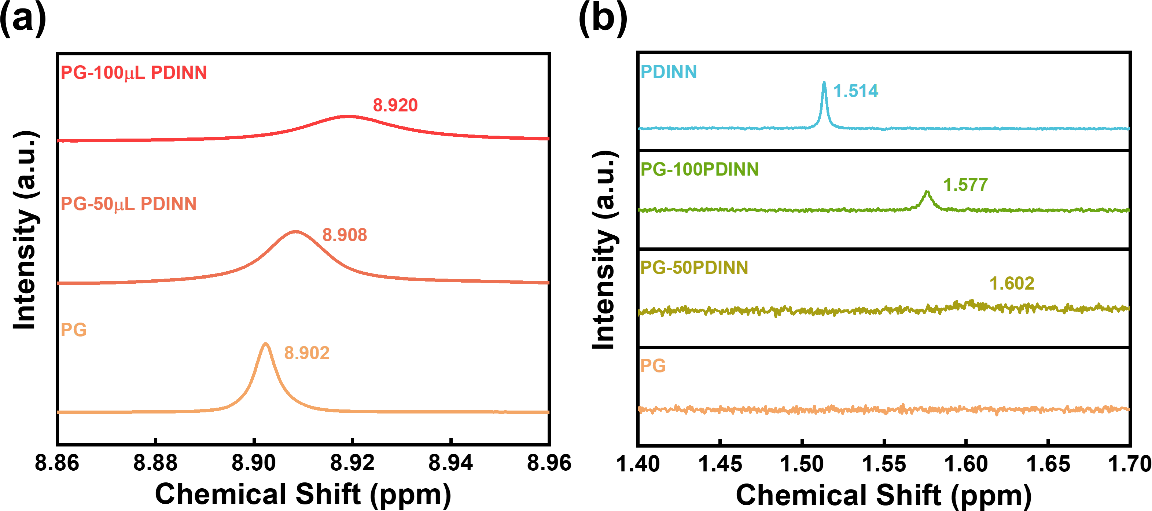
**

**Figure S3.** (a) and (b) Titration ^1^H NMR experiments conducted with PG and PDINN in solution of dimethyl sulfoxide-*d*6.

**6. Conductivity of CILs**


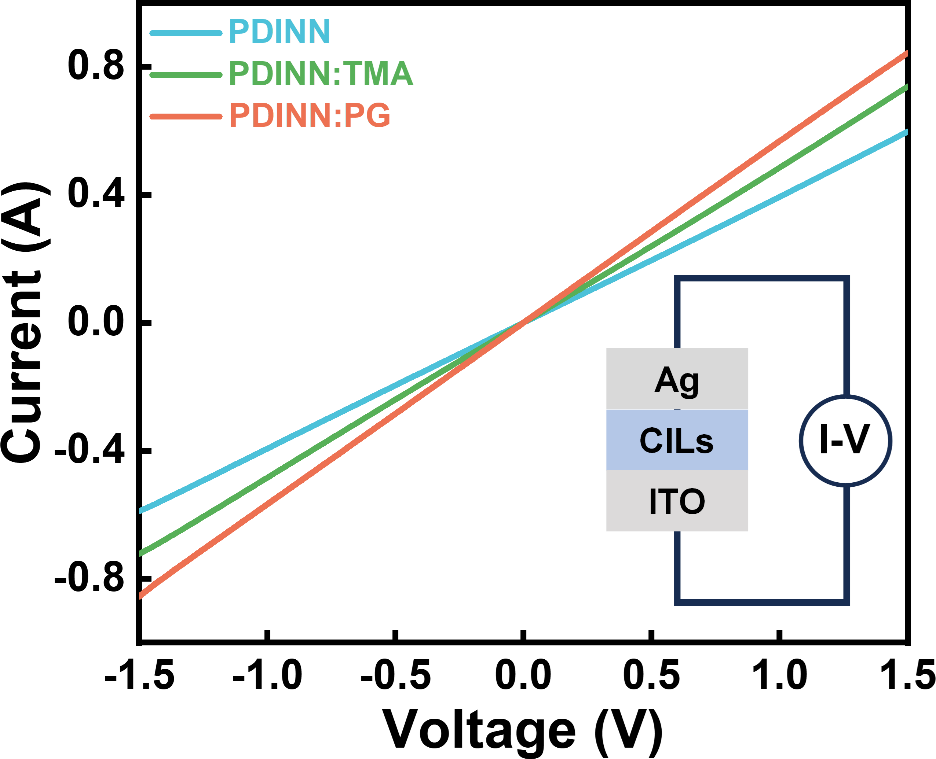


**Figure S4.** I–V curves of the devices with the structure of ITO/CILs/Ag.

**Table S1.** Electrical conductivity values for PDINN, PDINN:TMA, and PDINN:PG.

| **CILs** | **Conductivities(×10^-3^ mS/cm)** |
| --- | --- |
| PDINN | 3.94 |
| PDINN:TMA | 4.68 |
| PDINN:PG | 5.67 |

**7. UV-vis absorption spectra of Materials**


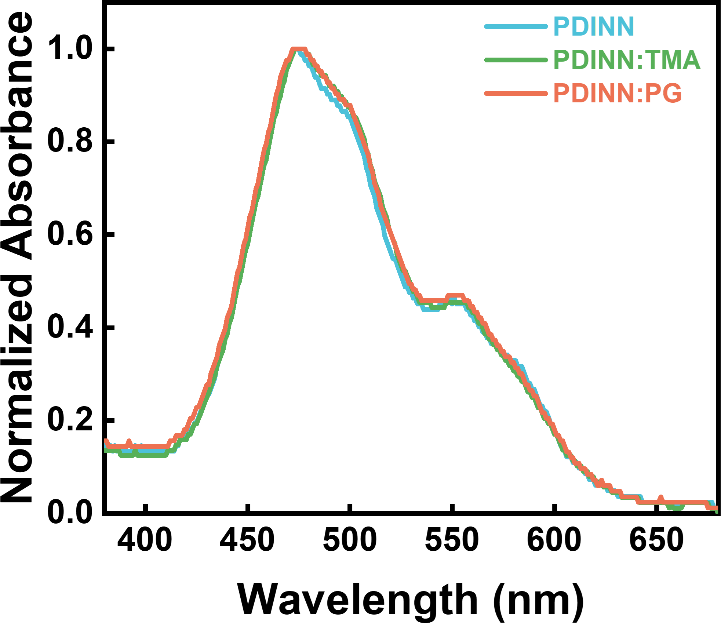


**Figure S5.** (a) UV–vis absorption spectra of PDINN, PDINN:TMA and PDINN:PG films.

**8. Energy level of Materials**

UPS spectra was measured by a Kratos Axis Ultra DLD spectrometer. The PDINN, PDINN:TMA, and PDINN:PG films were spin-coated on highly conductive ITO substrates. The UPS measurement was performed using a HeⅠ (*hv* = 21.22 eV) source, during which the vacuum of analysis chamber is maintained at 3.0×10^-8^ Torr and the applied bias voltage is -5 V. The work function (WF), the highest occupied molecular orbitals (HOMO) and the lowest unoccupied molecular orbitals (LUMO) energy levels can be separately obtained according to the following equations.

$WF= h\nu-E_{cutoff}$ (**1**)

$E_{HOMO}=h\nu-{(E}_{cutoff}-E_{onset})$ (**2**)

$E_{LUMO}=E_{HOMO}+E_{g}^{opt}$ (**3**)

where *hv* is the incident photon energy, equal to 21.22 eV; *E_cutof_*_f_ and *E_onset_* are the high binding energy cutoff and the low binding energy onset relative to the [Fermi level](https://www.sciencedirect.com/topics/engineering/fermi-level) (*E_F_*) of substrate, respectively, determined by the tangent extrapolation.^[2]^

**Table S2.** Comparison of the physicochemical properties of the CILs of PDINN, PDINN:TMA and PDINN:PG.

| **CIL** | **Work function**  **(eV)** | ***E^cutoff^* (eV)** | ***E^onset^* (eV)** | ***E^LUMO^* (eV)** | ***E_g_^opt^* (eV)** | ***E^HOMO^* (eV)** |
| --- | --- | --- | --- | --- | --- | --- |
| PDINN | 3.93 | 17.29 | 1.88 | -3.83 | 1.98 | -5.81 |
| PDINN:TMA | 3.88 | 17.33 | 1.96 | -3.86 | 1.98 | -5.84 |
| PDINN:PG | 3.82 | 17.40 | 2.08 | -3.92 | 1.98 | -5.90 |


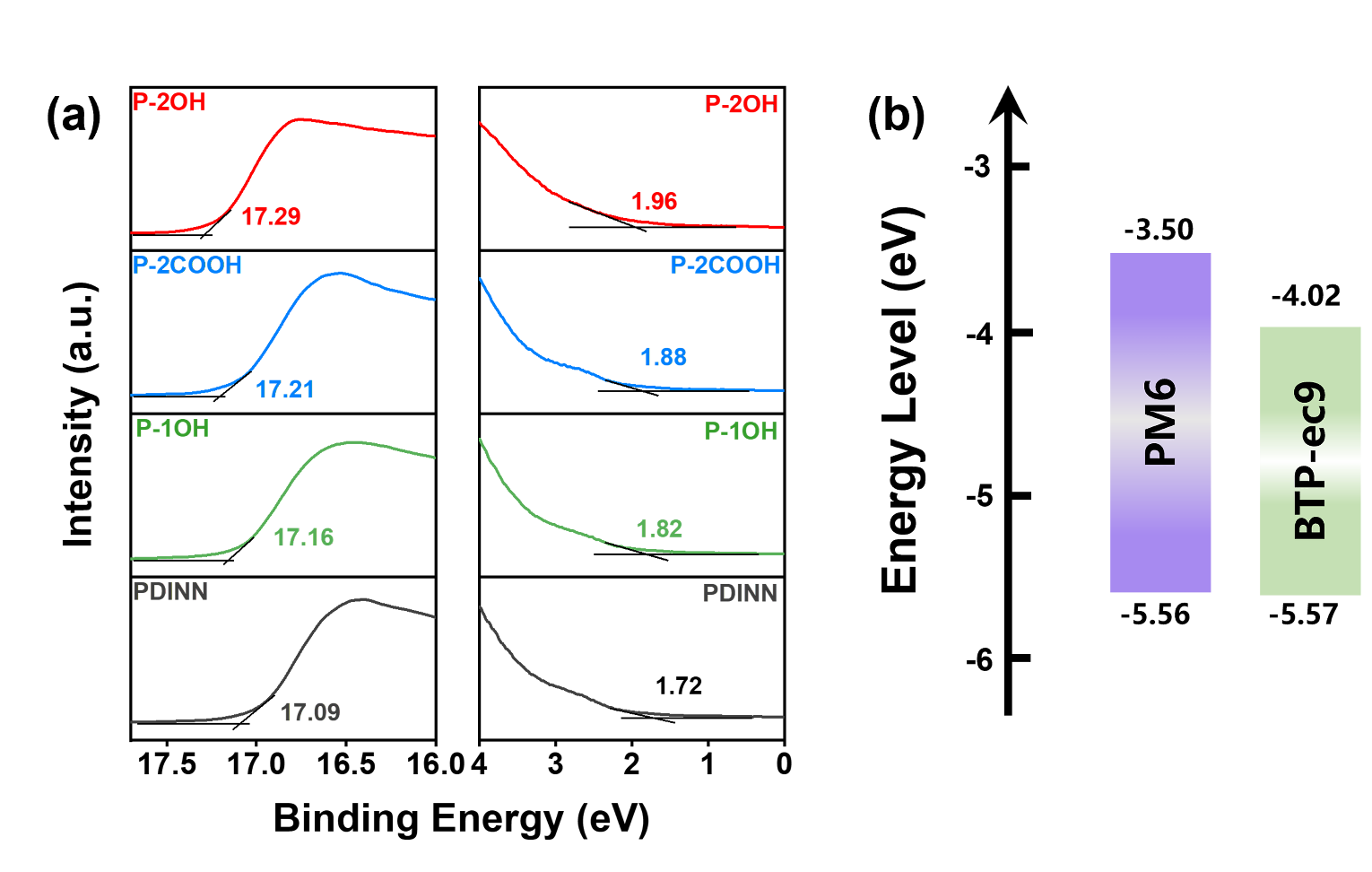


**Figure S6.** Energy level of PM6 and BTP-eC9.

**9. X-ray photoelectron spectroscopy (XPS) Measurements**


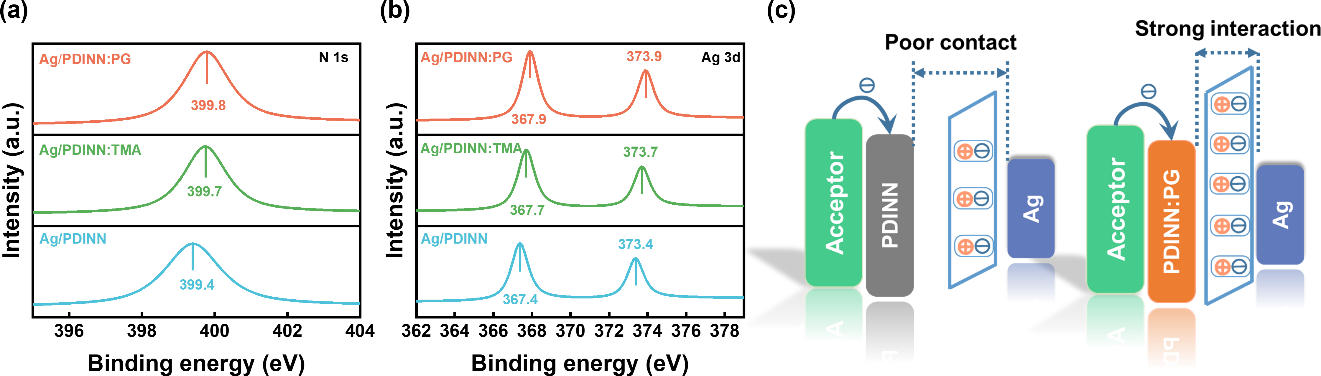


**Figure S7.** (a) X-ray photoelectron spectroscopy (XPS) spectra of N_1s_ on the Ag /PDINN, Ag/PDINN:TMA and Ag/PDINN:PG. (b) The spectrum of Ag 3d on the Ag /PDINN, Ag/PDINN:TMA and Ag/PDINN:PG surfaces. (c) Diagram of the electron extraction from devices with PDINN CIL and PDINN:PG CIL.

**10. Grazing Incidence Wide-angle X-ray Scattering (GIWAXS)**

GIWAXS measurements were performed at the Synchrotron & Printable Electronic Lab, Hoffmann Institute of Advanced Materials, Shenzhen Polytechnic University with SAXSFocus 3.0 (GKINST Co., LTD.) equipped with a Cu X-ray Source (8.05 keV, 1.54 Å) and an EIGER 2R 500K detector. The incident angle during the measurement was maintained at 0.5° and the distance between sample and detector was 132 mm. One dimensional experimental data were obtained with the SGTools software package programmed by Zhao et al.^[3]^

**Data analysis.** Structural parameters of blend films, including the periodicity of molecular arrangement and lamellar stacking spacing, were determined using the Bragg equation, while the crystal coherence length (*CCL*) can be obtained from the Scherrer formula. The corresponding expressions of the Bragg equation and Scherrer formula are as follows^[4]^:

$d=\frac{\lambda}{2\sin\left( \theta\right)}=\frac{2\pi}{q}$ (4)

$CCL=\frac{K\lambda}{FWHM\cdot\cos\left( \theta\right)}$ (5)

where *d* is the lamellar stacking spacing, and *CCL* is the crystal domain along the specified direction called crystal coherence length, which is generally considered to be equivalent to the grain size. 𝜆 is the value of X-ray wavelength; *K* is a dimensionless shape factor, generally taken as *K* = 0.89; FWHM is the half-peak width of the scattering peak; *θ* is the scattering angle.

**Table S3.** Detailed GIWAXS peak information IP of PDINN, PDINN:TMA and PDINN:PG blend film.

| **Component** | **Peak** | **Peak location (Å^−1^)** | **FWHM (Å^−1^)** | **d-spacing (Å)** | **Crystal coherence length (nm)** |
| --- | --- | --- | --- | --- | --- |
| PDINN | lamellar | 0.149 | 0.135 | 42.15 | 4.19 |
|  | lamellar | 0.347 | 0.123 | 18.10 | 4.60 |
|  | lamellar | 0.694 | 0.0864 | 9.05 | 6.54 |
|  | π–π stacking | 1.923 | 0.254 | 3.27 | 22.25 |
| PDINN:TMA | lamellar | 0.152 | 0.105 | 41.32 | 5.38 |
|  | lamellar | 0.348 | 0.126 | 18.05 | 4.49 |
|  | lamellar | 0.682 | 0.106 | 9.21 | 5.33 |
|  | π–π stacking | 1.928 | 0.234 | 3.26 | 24.05 |
| PDINN:PG | lamellar | 0.184 | 0.0983 | 34.13 | 5.75 |
|  | lamellar | 0.561 | 0.069 | 11.19 | 8.19 |
|  | π–π stacking | 1.937 | 0.235 | 3.24 | 24.15 |

**11. Transmission Electron Microscopy (TEM) and Element Mapping Diagrams**

TEM studies were performed a Thermo Fischer (former FEI) Titan 80-300 TEM equipped with an electron monochromator and a Gatan Imaging Filter (GIF) Quantum 966.


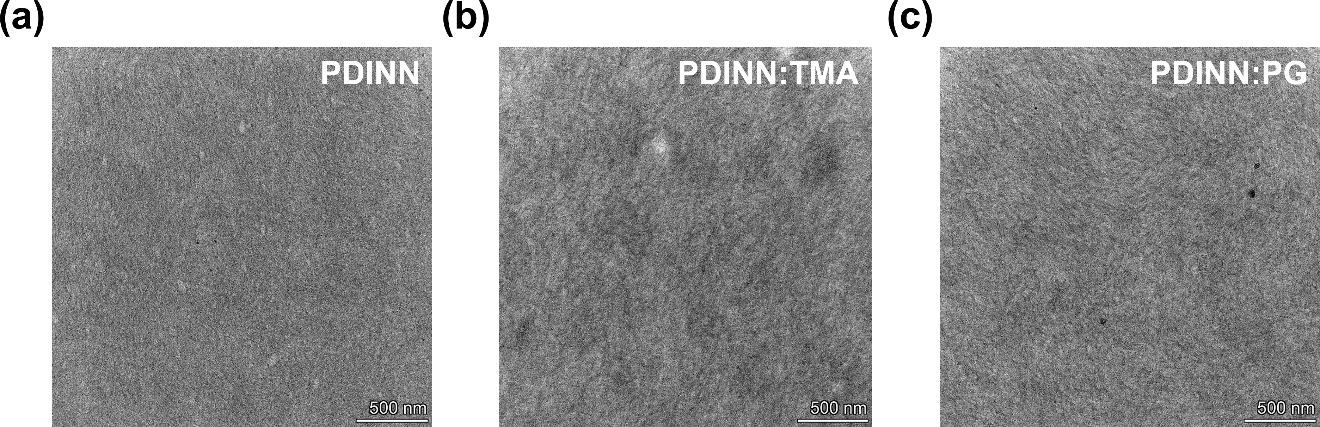


**Figure S8.** TEM images of PDINN, PDINN:TMA and PDINN:PG films.

1. **Atomic Force Microscopy (AFM) and** **Kelvin Probe Force Microscopy (KPFM)**

Topographic images of the films were obtained from a Bruker atomic force microscopy (AFM) with the type of dimension edge with Scan Asyst in the tapping mode using an etched silicon cantilever at a nominal load of ~2nN, the scanning rate for a 1 μm×1 μm image size was 0.9 Hz and 5 μm×5 μm image size was 1.0 Hz.

The Kelvin probe force microscopy (KPFM), The electric potential test parameters are set to Driver Frequency is 61.29144 kHz and Driver Amplitude is 500 mV. And the setting of Driver Routing is Driver Routing to Tip, so the position of high potential in the tested potential graph is the position of high sample potential and low work function.


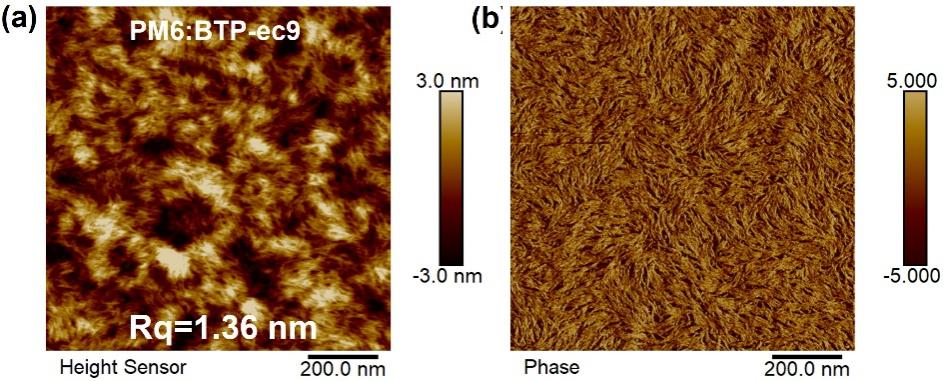


**Figure S9.** AFM image (a) and AFM phase (b) of PM6: BTP-eC9 film.

**13. Contact Angle Measurements**

The contact angles of water and formamide (FA) on SAMs coated ITO were measured by a video optical contact angle meter (DSA-100 (KRUSS Germany)). Then the surface free energy was calculated by Owens-Wendt method: ^[5]^

$\gamma_{L}\times\left( 1+cos\theta\right)=2 \times\left( \gamma_{L}^{d}\cdot\gamma_{sv}^{d} \right)^{\frac{1}{2}}+2\times\left( \gamma_{L}^{p}\cdot\gamma_{sv}^{p} \right)^{\frac{1}{2}}$ (**9**)

where $\gamma_{L}$ and $\gamma_{sv}$ are surface free energy of the probe liquid and sample, respectively. The $\theta$ is the contact $\gamma_{L}$ angle of the sample. The Flory-Huggins interaction parameter *χ*_donor-acceptor_ for blends to show the binary miscibility was calculated from the equation:

$\chi_{door-acceptor}=K\left( \gamma_{donor}^{\frac{1}{2}}-\gamma_{donor}^{\frac{1}{2}} \right)^{2}$ (**10**)

where γ is the surface energy of the material, *K* is the proportionality constant.

**Table S4.** Summary of the surface energies for the various surfaces.

| **Surface** | **Surface energy (mN/m)** |
| --- | --- |
| PM6: BTP-eC9 | 11.53 |
| PDINN | 68.50 |
| PDINN:TMA | 68.30 |
| PDINN:PG | 68.24 |


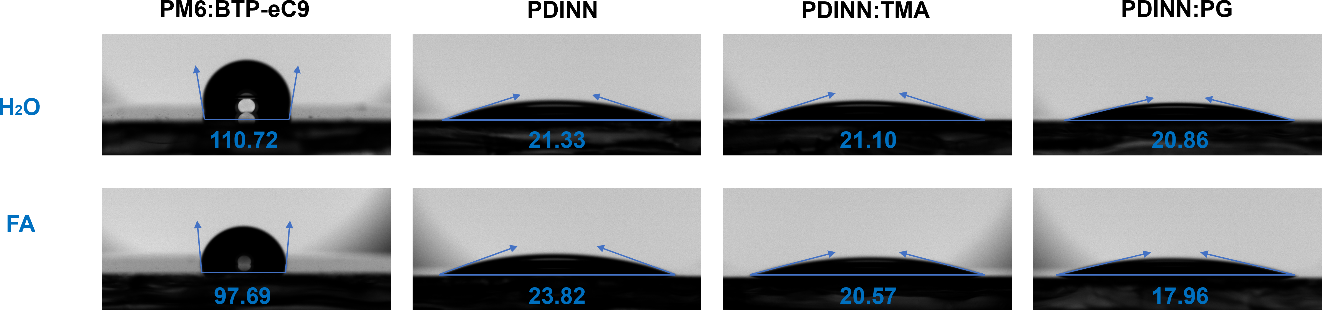


**Figure S10.** Photographs of water (top row) and formamide (bottom row) droplets in contact with the various layer surfaces.

**14. OSCs Device Performance Data**

**Table S5.** The photovoltaic data of the OSCs based on PM6: BTP-eC9 with PDINN:TMA and PDINN:PG CILs of different conditions, and the device structure is ITO/2Br-2PACZ/PM6: BTP-eC9/CILs/Ag. All data were obtained under AM 1.5G (100 mW/cm^2^) illumination in conventional devices.

|  | **Concentration/(mg/mL)** | ***V_OC_* (V)** | ***J_SC_* (mA/cm^2^)** | **FF (%)** | **PCE (%)** |
| --- | --- | --- | --- | --- | --- |
| TMA | 0.05 | 0.858  (0.857±0.001) | 27.60  (27.44±0.24) | 78.0  (78.0±0.2) | 18.5  (18.3±0.2) |
|  | 0.10 | 0.859  (0.859±0.002) | 28.09  (28.06±0.15) | 78.2  (77.6±0.4) | 18.9  (18.7±0.1) |
|  | 0.20 | 0.859  (0.859±0.001) | 28.05  (27.84±0.16) | 77.2  (77.2±0.2) | 18.6  (18.5±0.1) |
| PG | 0.05 | 0.863  (0.863±0.001) | 28.10  (28.07±0.09) | 78.7  (78.5±0.1) | 19.1  (19.0±0.1) |
|  | 0.10 | 0.864  (0.863±0.001) | 28.57  (28.47±0.18) | 79.2  (78.8±0.2) | 19.6  (19.4±0.2) |
|  | 0.20 | 0.861  (0.861±0.001) | 28.36  (28.23±0.40) | 78.4  (78.3±0.3) | 19.2  (19.0±0.3) |


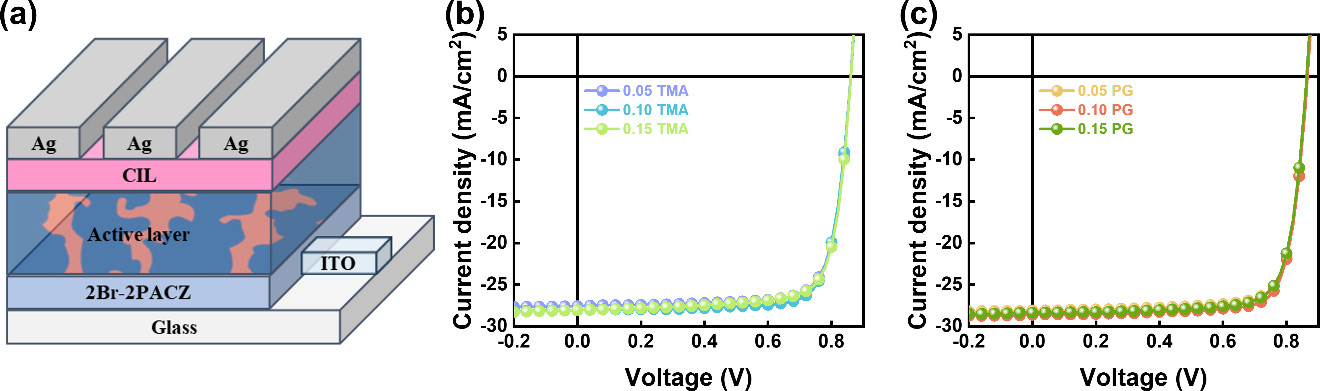


**Figure S11.** (a) The device of conventional architecture. *J-V* curves of the OSCs based on PM6: BTP-eC9 with (b) PDINN:TMA and (c) PDINN:PG CILs of different conditions.


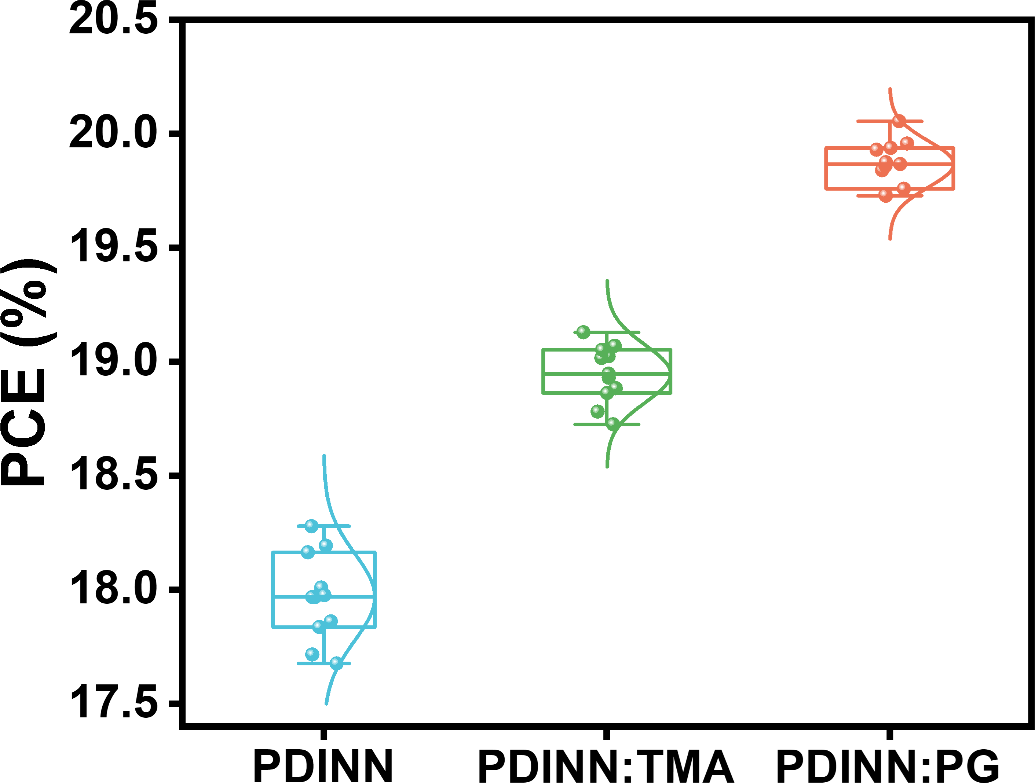


**Figure S12.** Statistical PCE distribution of three devices.


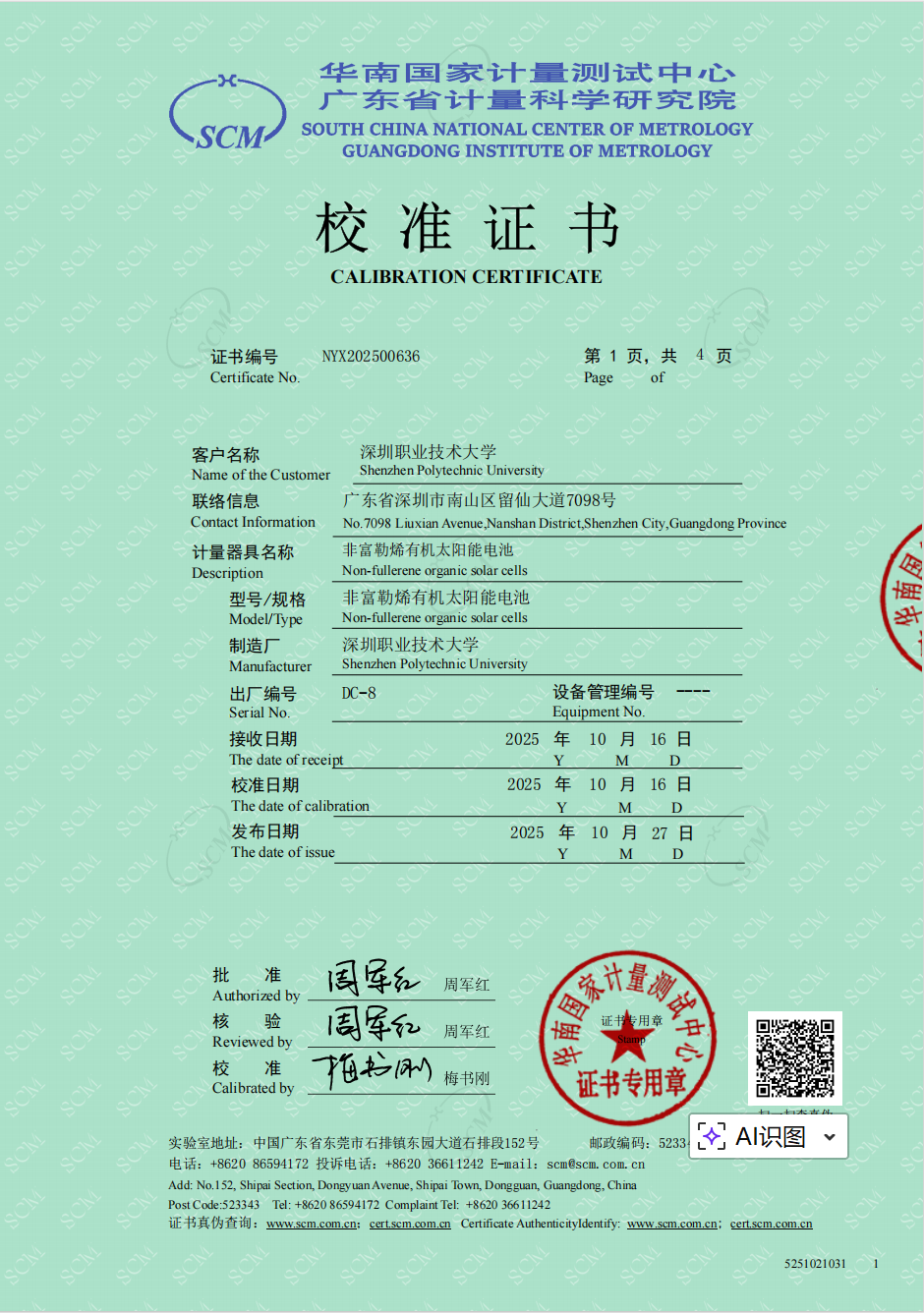


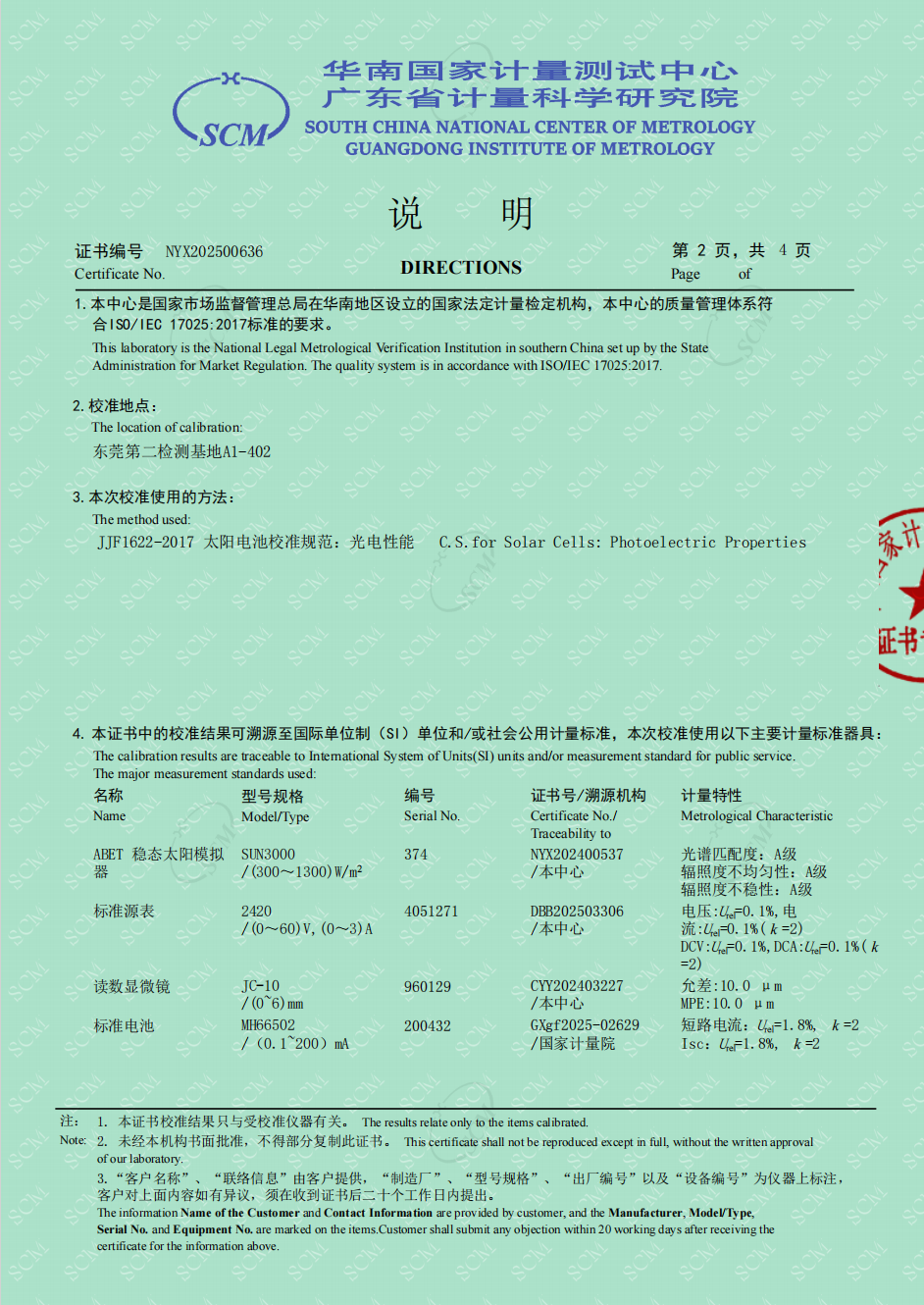


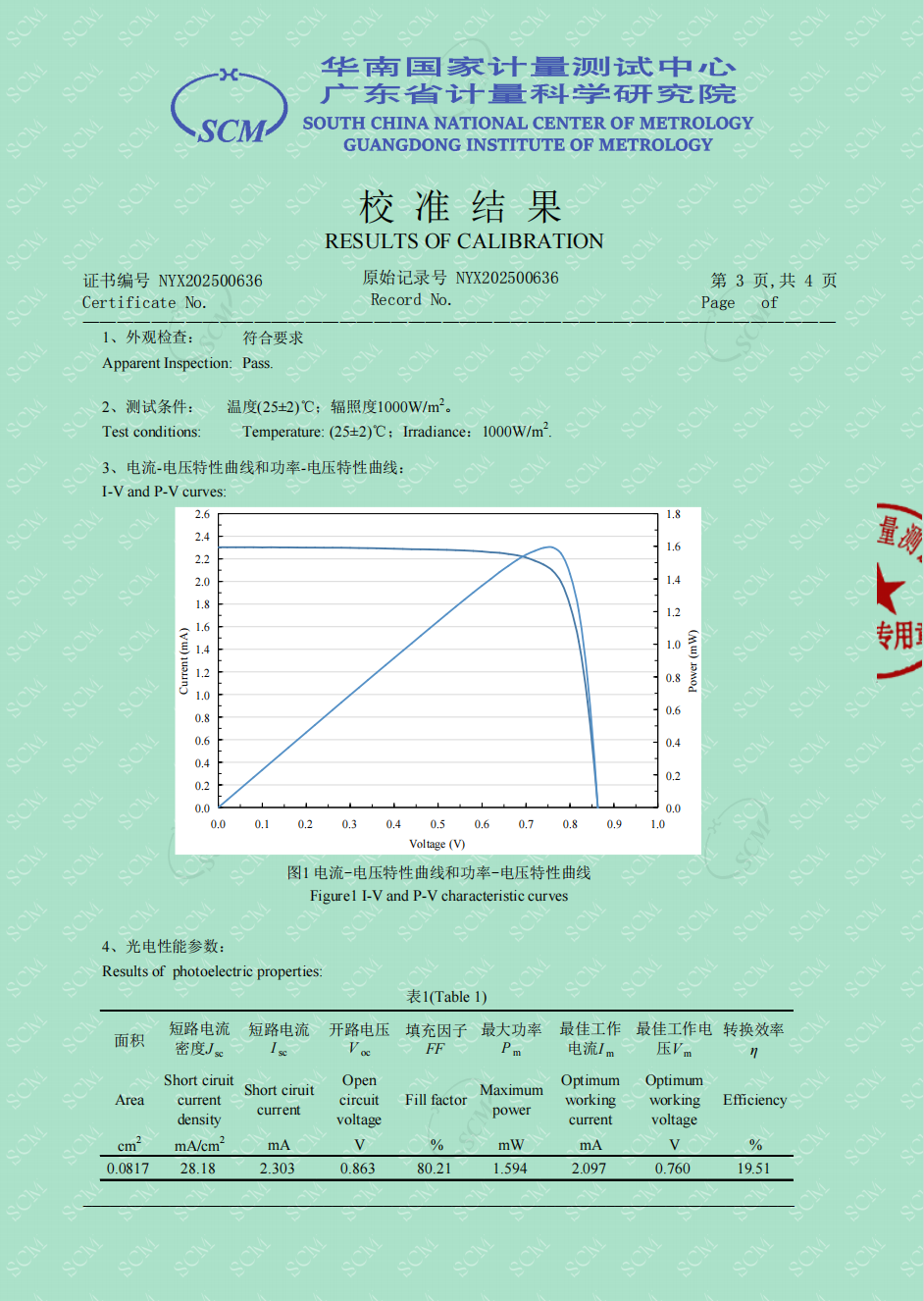


**Figure S13.** Certified Efficiency of PM6: BTP-eC9 binary OSCs device based on PDINN:PG-CIL.

**15. Exciton dissociation data**

**Table S6.** The PDINN, PDINN:TMA and PDINN:PG devices corresponding parameters of exciton dissociation efficiency (η_diss_ = *J_SC_* / *J_sat_*) and charge collection efficiency (η_coll_ = *J_max power_* / *J_sat_*).^[6]^

| **Parameter** | **PDINN** | **PDINN:TMA** | **PDINN:PG** |
| --- | --- | --- | --- |
| *J_sat_* (mA/cm^2^) | 27.64 | 28.53 | 28.92 |
| *J_SC_* (mA/cm^2^) | 27.12 | 28.04 | 28.47 |
| η_diss_ = *J_SC_* / *J_sat_* (%) | 98.09 | 98.22 | 98.43 |
| *J_max_* (mA/cm^2^) | 24.39 | 25.74 | 26.43 |
| η_coll_ = *J_max_ _power_* / *J_sat_* (%) | 88.23 | 90.21 | 91.36 |

**16. Electrochemical Impedance Spectroscopy (EIS)**


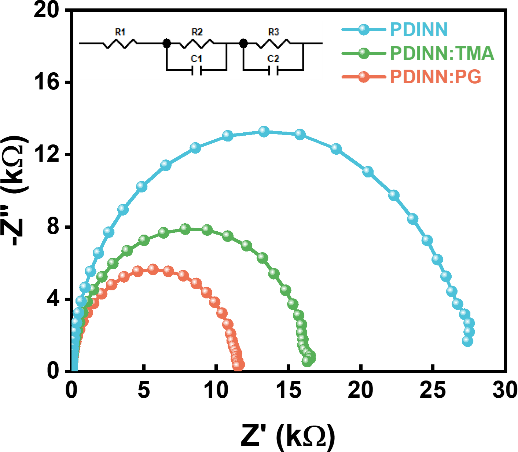


**Figure S14.** Nyquist plots of devices based on PDINN, PDINN:TMA, and PDINN:PG CILs in the dark.

**17. SCLC Mobility Measurements and Trap Density**

Fitting the hole/electron-only diode dark current to the space charge limited current (SCLC) model can obtain the hole and electron mobility of the photosensitive active layer. The electron-only device structure was ITO/ZnO/PDINN/Active layer/CILs/Ag and the hole-only device structure was ITO/PEDOT: PSS/Active layer/MoO_3_/Ag. Using the following equation to estimate the electric-field dependent SCLC mobility:

$J\left( V \right)=\frac{9}{8}\varepsilon_{0}\varepsilon_{r}\mu_{0}exp\left( 0.89\beta\sqrt{\frac{V-V_{bi}}{L}} \right)\frac{{(V-V_{bi})}^{2}}{L^{3}}$ (**4**)

For the hole-only device structure, V_bi_ = 0 V (flat band pattern formed by PEDOT: PSS-MoO_3_); For the electron-only device structure, V_bi_ = 0.5 V was used following the protocol reported.^[7-8]^

The trap density is extracted from the logarithmic slope curve of the *J*-V characteristic of the electron-only device, using equation:

$N_{trap}=\frac{2\varepsilon_{0}\varepsilon_{r}V_{TFL}}{qL^{2}}$ (**5**)

Where $\varepsilon_{0}$ is the permittivity of free space, $\varepsilon_{r}$ is the dielectric constant of the active layer (assumed to be 3 in this calculation), *V_TFL_* is the peak location of the logarithmic slope curve, *q* refers to the element charge and *L* is the thickness of the active layer.^[9]^


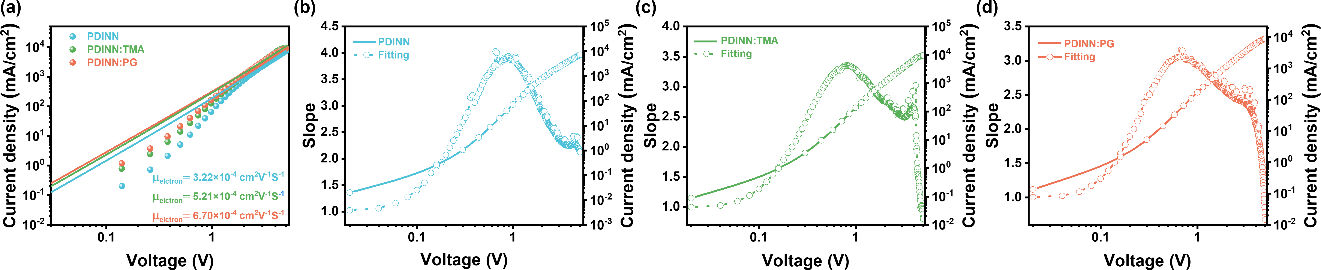


**Figure S15.** (a) Dark *J-V* curves of the OSCs: electron-only diodes, the solid lines are fit to the experimental data according to SCLC model. *J-V* curves and the corresponding logarithmic slope curves of the electron-only devices with (b) PDINN, (c) PDINN:TMA and (d) PDINN:PG CILs.

**Table S7.** Electron mobilities (µ_electron_) and extracted trap density of PDINN, PDINN:TMA and PDINN:PG devices.

| **CIL** | **µ_electron_(×10^-4^cm^2^V^-1^S^-1^)** | ***V_TFL_* (V)** | **Trap Density (/cm^3^)** |
| --- | --- | --- | --- |
| PDINN | 3.22 | 0.817 | 2.87×10^18^ |
| PDINN:TMA | 5.21 | 0.763 | 2.68×10^18^ |
| PDINN:PG | 6.70 | 0.671 | 2.36×10^18^ |

**18. Energy Losses (E_loss_)**

Specification of the sources of Eloss follows the equation:^[10]^

$E_{\mathrm{LOSS}}=\left( E_{g}-\mathrm{qV}_{\mathrm{OC}}^{\mathrm{SQ}} \right)+\mathrm{qV}_{\mathrm{OC}}^{rad,below gap}+\mathrm{qV}_{\mathrm{OC}}^{non-rad}=\Delta E_{1}+\Delta E_{2}+\Delta E_{3}$ (**6**)

where E_g_ is the band-gap, V_OC_^SQ^ is the maximum V_OC_ under the S-Q limit, and V_OC_^rad^ is the V_OC_ when only radiative recombination is considered. The final part of the non-radiative recombination loss (∆E_3_) is obtained by the calculation equation:^[11]^

$\Delta E_{3}=\mathrm{qV}_{\mathrm{OC}}^{non-rad}=-kTln(\mathrm{EQE}_{EL})$ (**7**)

where k is the Boltzmann constant, T is absolute temperature.

For disordered semiconductors that generally produce localized states extending into the bandgap, the optical absorption coefficient (*α*(*E*)) in the low photon energy range follows the Urbach rule following:^[12]^

α(E) = α_0_ e^(E-Eg)/Eu^ (**8**)

where α_0_ is the optical absorption coefficient at the band edge, E is the photon energy and Eu is the Urbach energy.


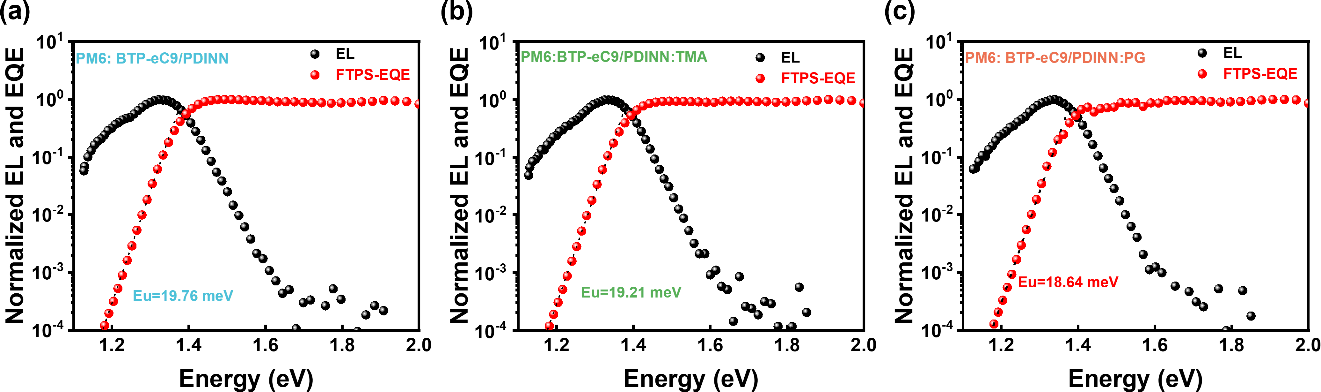


**Figure S16.** Normalized EL and FTPS-EQE spectra of (a) PDINN, (b) PDINN:TMA and (c) PDINN:PG devices.

**Table S8.** Detailed energy losses in devices based on PM6: BTP-eC9 with different CILs.

| **CIL** | **ΔE_1_** | **ΔE_2_** | **ΔE_3_** | **E_loss_** |
| --- | --- | --- | --- | --- |
| PDINN | 0.26 | 0.057 | 0.237 | 0.554 |
| PDINN:TMA | 0.26 | 0.051 | 0.229 | 0.540 |
| PDINN:PG | 0.26 | 0.051 | 0.217 | 0.528 |

**19. Photovoltaic Performance Data of OSCs**

**Table S9.** Photovoltaic Performance of the OSCs with different PDINN and PDINN:PG CILs thickness under the illumination of AM 1.5G 100 mW/cm^2^.

| **CIL Thickness (nm)** | | ***V_OC_* (V)** | ***J_SC_* (mA/cm^2^)** | **FF (%)** | **PCE (%)** |
| --- | --- | --- | --- | --- | --- |
| 10 | PDINN | 0.857  (0.856±0.002) | 27.58  (27.22±0.18) | 77.4  (77.1±0.4) | 18.3  (18.0±0.2) |
| 15 |  | 0.852  (0.850±0.003) | 26.95  (26.67±0.19) | 75.1  (75.0±0.4) | 17.3  (17.0±0.2) |
| 20 |  | 0.840  (0.840±0.002) | 26.72  (26.32±0.42) | 73.3  (73.3±0.6) | 16.5  (16.2±0.2) |
| 25 |  | 0.831  (0.829±0.003) | 26.32  (26.08±0.34) | 72.5  (72.3±0.6) | 15.9  (15.6±0.3) |
| 30 |  | 0.823  (0.822±0.004) | 26.08  (25.60±0.45) | 72.3  (72.3±0.3) | 15.5  (15.2±0.3) |
| 50 |  | 0.794  (0.781±0.015) | 24.72  (24.66±0.38) | 70.2  (70.0±0.5) | 13.8  (13.5±0.2) |
| 10 | PDINN:PG | 0.863  (0.863±0.001) | 28.82  (28.69±0.11) | 80.6  (80.2±0.3) | 20.0  (19.9±0.1) |
| 15 |  | 0.864  (0.864±0.001) | 28.36  (27.55±0.10) | 77.9  (77.7±0.2) | 19.1  (18.9±0.2) |
| 20 |  | 0.864  (0.862±0.001) | 28.25  (28.24±0.11) | 76.7  (76.3±0.6) | 18.7  (18.6±0.1) |
| 25 |  | 0.864  (0.862±0.003) | 27.77  (27.57±0.19) | 76.4  (76.4±0.2) | 18.3  (18.2±0.1) |
| 30 |  | 0.863  (0.863±0.003) | 27.09  (26.96±0.20) | 76.4  (76.1±0.3) | 17.9  (17.6±0.2) |
| 50 |  | 0.857  (0.853±0.003) | 26.98  (26.79±0.21) | 75.2  (75.2±0.4) | 17.4  (17.2±0.2) |

**Table S10.** Photovoltaic Performance of the OSCs with different PDINN and PDINN:PG CILs thickness under the illumination of AM 1.5G 100 mW/cm^2^.

| **Active layer Thickness (nm)** | **CILs** | ***V_OC_* (V)** | ***J_SC_* (mA/cm^2^)** | **FF (%)** | **PCE (%)** |
| --- | --- | --- | --- | --- | --- |
| 100nm | PDINN | 0.857  (0.856±0.002) | 27.58  (27.22±0.18) | 77.4  (77.1±0.4) | 18.3  (18.0±0.2) |
| 200nm |  | 0.847  (0.847±0.002) | 27.78  (27.66±0.20) | 71.4  (71.0±0.8) | 16.8  (16.6±0.2) |
| 300nm |  | 0.847  (0.847±0.015) | 28.49  (28.42±0.11) | 65.2  (65.2±0.1) | 15.7  (15.7±0.1) |
| 100nm | PDINN:PG | 0.863  (0.863±0.001) | 28.82  (28.69±0.11) | 80.6  (80.2±0.3) | 20.0  (19.9±0.1) |
| 200nm |  | 0.856  (0.855±0.001) | 28.54  (28.28±0.42) | 74.4  (74.4±0.1) | 18.2  (18.0±0.3) |
| 300nm |  | 0.852  (0.851±0.001) | 29.78  (29.73±0.21) | 69.5  (69.3±0.2) | 17.6  (17.5±0.1) |

**
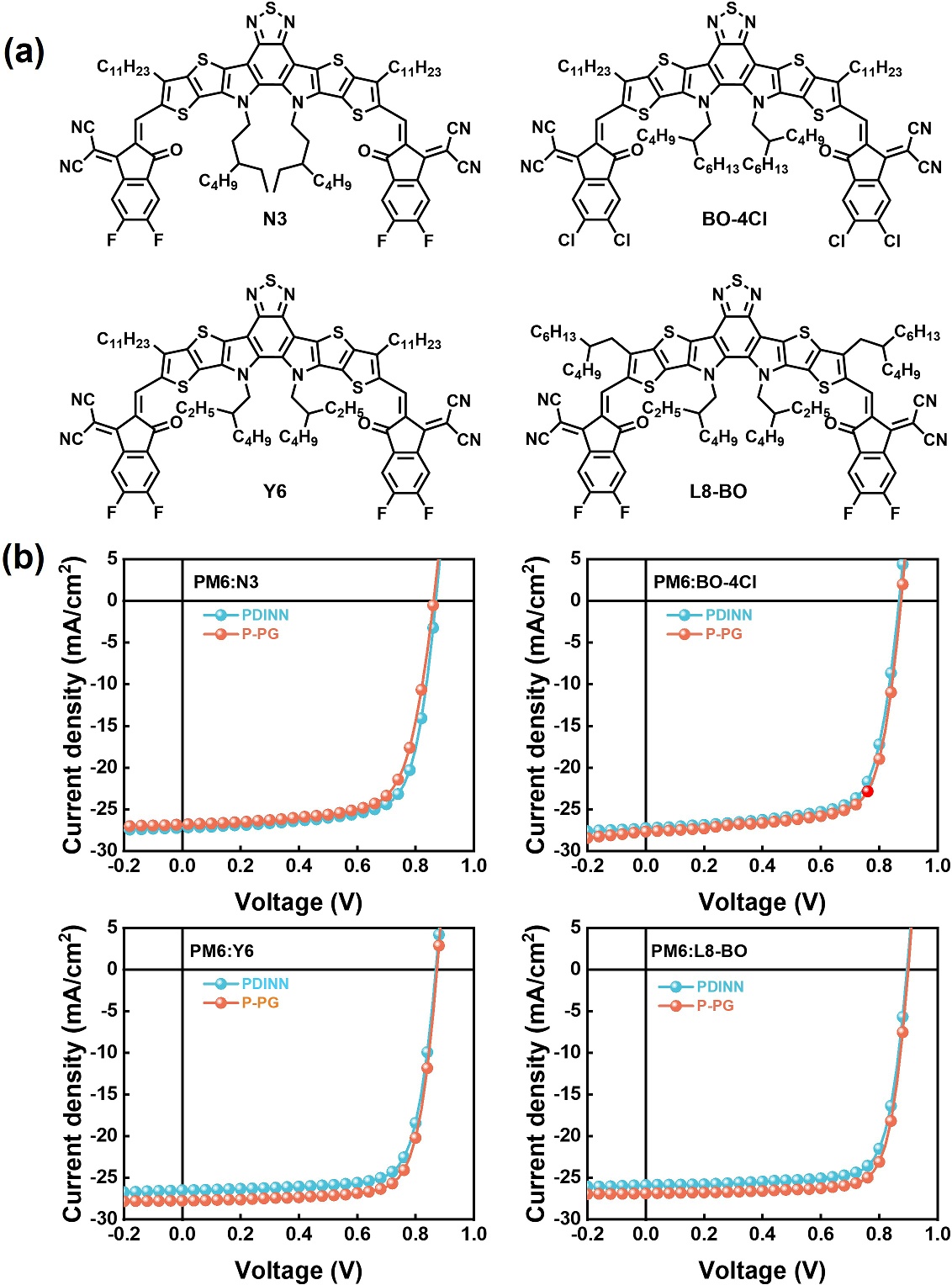
**

**Figure S17.** (a) Molecular structures of N3, BO-4Cl, Y6 and L8-BO. (b) *J-V* curves of the OSCs based on PM6: donor with PDINN and PDINN:PG CILs.

**Table S11.** The photovoltaic data of the OSCs based on PM6 donor with PDINN and PDINN:PG CILs under the AM 1.5G (100 mW/cm^2^) light source illumination. And the device structure is ITO/2Br-2PACz/PM6: donor /CIL/Ag.

| **Active Layer** | **CIL** | ***V_OC_* (V)** | ***J_SC_* (mA/cm^2^)** | **FF (%)** | **^b^PCE (%)** |
| --- | --- | --- | --- | --- | --- |
| PM6: Y6 | PDINN | 0.868  (0.867±0.001) | 26.51  (26.48±0.11) | 75.7  (75.4±0.2) | 17.4  (17.3±0.1) |
|  | PDINN:PG | 0.872  (0.870±0.001) | 27.76  (27.74±0.03) | 76.3  (76.1±0.1) | 18.5  (18.4±0.1) |
| PM6:BO-4Cl | PDINN | 0.867  (0.865±0.001) | 27.27  (26.93±0.45) | 71.81  (71.43±0.25) | 16.97  (16.65±0.33) |
|  | PDINN:PG | 0.873  (0.870±0.002) | 27.66  (27.53±0.20) | 72.58  (72.39±0.56) | 17.53  (17.33±0.12) |
| PM6: N3 | PDINN | 0.861  (0.861±0.002) | 26.81  (26.66±0.44) | 70.6  (70.1±0.3) | 16.3  (16.2±0.2) |
|  | PDINN:PG | 0.868  (0.866±0.002) | 27.19  (27.17±0.10) | 72.7  (72.1±0.4) | 17.1  (17.0±0.1) |
| PM6: L8-BO | PDINN | 0.894  (0.893±0.001) | 25.89  (25.85±0.06) | 77.1  (76.9±0.3) | 17.8  (17.6±0.2) |
|  | PDINN:PG | 0.898  (0.897±0.001) | 26.88  (26.70±0.12) | 78.34  (78.51±0.2) | 18.5  (18.3±0.2) |


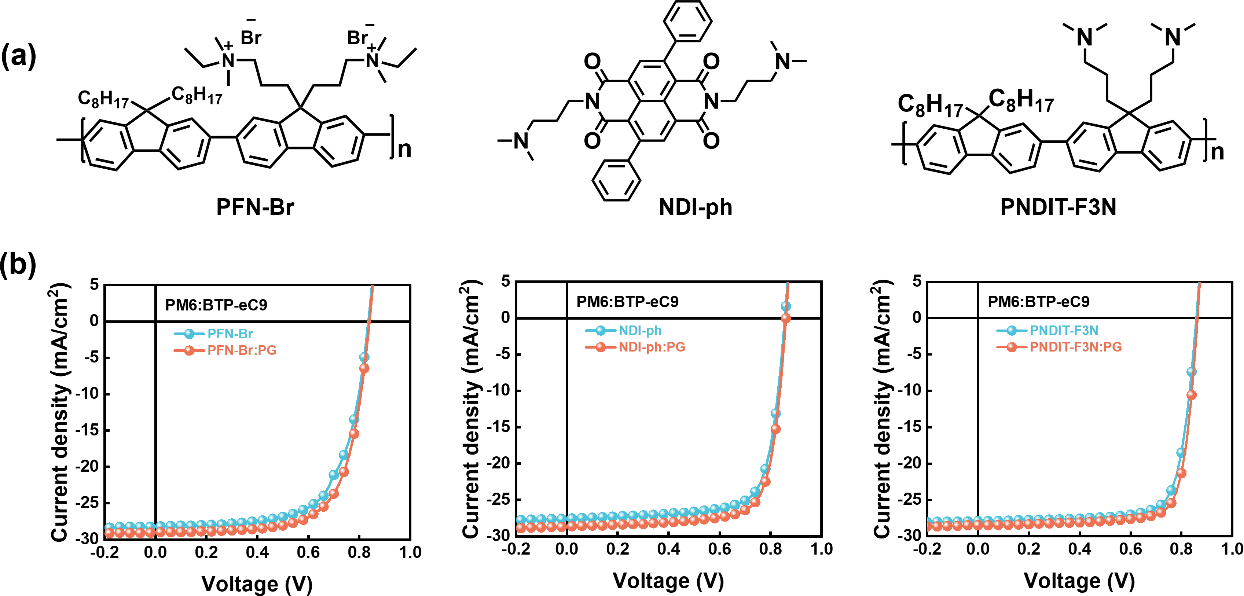


**Figure S18.** (a) Molecular structures of PFN-Br, NDI-ph and PNDIT-F3N. (b) *J-V* curves of the OSCs based on PM6: BTP-eC9 with CILs and CILs-PG.

**Table S12.** The photovoltaic data of the OSCs based on PM6: BTP-eC9 with different CILs under PG regulation under the AM 1.5G (100 mW/cm^2^) light source illumination, and the device structure is ITO/2Br-2PACZ/PM6: BTP-eC9/CIL/Ag.

| **Active Layer** | **CIL** | ***V_OC_* (V)** | ***J_SC_* (mA/cm^2^)** | **FF (%)** | **^b^PCE (%)** |
| --- | --- | --- | --- | --- | --- |
| PM6: BTP-eC9 | PFN-Br | 0.834  (0.834±0.001) | 28.21  (27.90±0.32) | 66.8  (66.7±0.3) | 15.7  (15.5±0.13) |
|  | PFN-Br -PG | 0.837  (0.837±0.001) | 29.02  (29.02±0.35) | 68.9  (67.2±0.7) | 16.8  (16.3±0.2) |
|  | NDI-ph | 0.855  (0.853±0.001) | 27.58  (27.36±0.22) | 74.9  (72.5±0.2) | 17.7  (17.4±0.2) |
|  | NDI-ph-PG | 0.858  (0.856±0.002) | 28.66  (28.31±0.23) | 75.80  (74.9±0.3) | 18.7  (18.2±0.2) |
|  | PNDIT-F3N | 0.857  (0.857±0.001) | 27.92  (27.55±0.38) | 76.9  (76.5±0.5) | 18.4  (18.1±0.2) |
|  | PNDIT-F3N-PG | 0.862  (0.861±0.001) | 28.47  (28.27±0.25) | 78.9  (78.4±0.4) | 19.3  (19.1±0.2) |

**20. The Maximum Power Point (MPP) tracking Measurements**

The operational stability was performed using a stability setup. (MPP Tracking-4B, who's with the range ±2.4V for voltage and ±10mA for Current), tested under continuous light illumination and maximum power point tracking. The light source consisted of an array of white LEDs powered by a constant current. The LED type is·SLS-LED-80A with an emission spectrum of 400-1000 nm.

**21. The External Quantum Efficiency (EQE) date of TSCs**


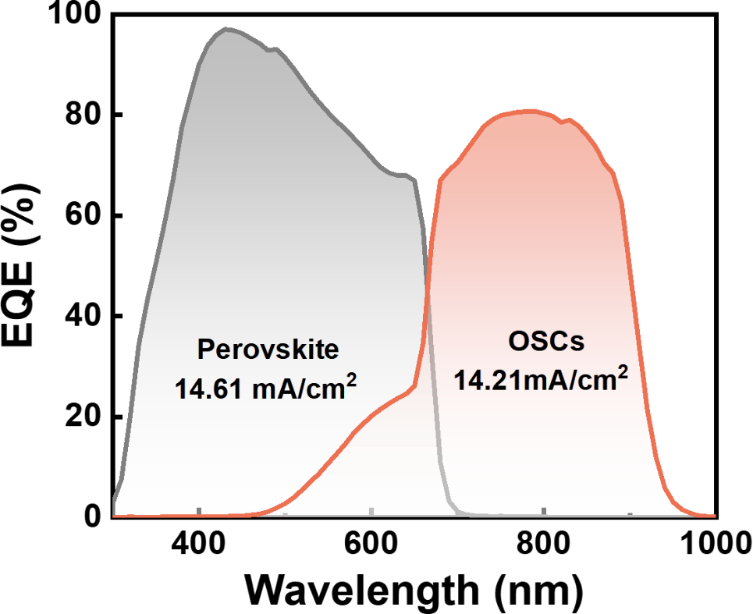


**Figure S19.** EQE curves of TSCs.

**22. References**

[1] S. Xiong, L. Hu, L. Hu, et al., “12.5% Flexible Nonfullerene Solar Cells by Passivating the Chemical Interaction Between the Active Layer and Polymer Interfacial Layer,” *Advanced Materials* **31** (2019): 1806616, <https://doi.org/10.1002/adma.201806616>.

[2] Z. A. Page, Y. Liu, V. V. Duzhko, et al., “Fulleropyrrolidine interlayers: Tailoring electrodes to raise organic solar cell efficiency,” *Science* **346** (2014): 441, https://doi. org/[10.1126/science.125582](https://doi.org/10.1126/science.1255826).

[3] N. Zhao, C. Yang, F. Bian, et al., “SGTools: a suite of tools for processing and analyzing large data sets from in situ X-ray scattering experiments,” *Journal of applied crystallography* **55** (2022): 195, [https://doi.org/10.1107/S1600576721012267](https://doi.org/10.1107/S1600576721012267).

[4] X. Sun, C. Zhang, Y. Yao, et al., “19.35% Efficient Binary Bulk-Heterojunction Organic Photovoltaic Enabled by Optimizing Bromine-Substituted Self-Assembled Carbazole Based Molecules,” *Advanced Functional Materials* **34** (2024): 2406060, <https://doi.org/10.1002/adfm.202406060>.

[5] A. Rudawska, E. Jacniacka, “Analysis for determining surface free energy uncertainty by the Owen–Wendt method,” *International Journal of Adhesion and Adhesives* **29** (2009): 451, [https://doi.org/10.1016/j.ijadhadh.2008.09.008](https://doi.org/10.1016/j.ijadhadh.2008.09.008).

[6] T. Xu, J. Lv, K. Yang, et al., “15.8% efficiency binary all-small-molecule organic solar cells enabled by a selenophene substituted sematic liquid crystalline donor,” *Energy & Environmental Science* **14** (2021): 5366, https://doi.org/10.1039/D1EE01193F.

[7] L. Hou, J. Lv, F. Wobben, et al., “Effects of Fluorination on Fused Ring Electron Acceptor for Active Layer Morphology, Exciton Dissociation, and Charge Recombination in Organic Solar Cells,” *ACS Applied Materials & Interfaces* **12** (2020): 56231, https://doi.org/10.1021/acsami.0c16411.

[8] K. Wang, J. Lv, T. Duan, et al., “Simple near-Infrared Nonfullerene Acceptors Enable Organic Solar Cells with >9% Efficiency,” *ACS Applied Materials & Interfaces* **11** (2019): 6717, [https://doi.org/10.1021/acsami.8b20567](https://doi.org/10.1021/acsami.8b20567).

[9] P. Bi, S. Zhang, T. Xiao, et al., “Suppressing trap states and energy loss by optimizing vertical phase distribution through ternary strategy in organic solar cells,” *Science China Chemistry* **64** (2021): 599, https://doi. org/10.1007/s11426-020-9926-x.

[10] X.-K. Chen, D. Qian, Y. Wang, et al., “A unified description of non-radiative voltage losses in organic solar cells,” *Nature Energy* **6** (2021): 799, https://doi.org/10.1038/s41560-021-00843-4.

[11] T. Fritsch, J. Kurpiers, S. Roland, et al., “On the Interplay between CT and Singlet Exciton Emission in Organic Solar Cells with Small Driving Force and Its Impact on Voltage Loss,” *Advanced Energy Materials* **12** (2022): 2200641, https://doi.org/10.1002/aenm.202200641.

[12] S. Liu, J. Yuan, W. Deng, et al., “High-efficiency organic solar cells with low non-radiative recombination loss and low energetic disorder,” *Nature Photonics* **14** (2020): 300, https://doi.org/10.1038/s41566-019-0573-5.
